# Double-hit approach for novel glycoconjugates combining cytoplasmic glycoengineering and selective chemistry

**DOI:** 10.1039/d5cb00320b

**Published:** 2026-01-15

**Authors:** Elena Palmieri, Valentina Monaci, Salvatore Durante, Paola Cescutti, Francesca Micoli, Gianmarco Gasperini

**Affiliations:** a GSK Vaccines Institute for Global Health (GVGH), Via Fiorentina 1 53100 Siena Italy; b Resonance Center – CERM, University of Florence, Via Luigi Sacconi 6, Sesto Fiorentino 50019 Florence Italy; c GSK, Via Fiorentina 1 53100 Siena Italy gianmarco.x.gasperini@gsk.com; d Department of Life Sciences, University of Trieste, Via L. Giorgieri 1, Bldg C11 34127 Trieste Italy

## Abstract

Chemical conjugation to carrier proteins has been traditionally used to improve polysaccharides immunogenicity and to overcome the limitations of T-independent antigens, including lack of immunological memory and efficacy in infants. A double-hit approach, meaning that both polysaccharide and carrier protein belong to the same pathogen, may be particularly useful for targeting bacterial species with large glycan variability. Recently, bacterial protein glycosylation has been exploited to obtain glycosylated proteins in *E. coli* cytoplasm. In our work we have combined cytoplasmic glycoengineering and chemical conjugation for the development of novel selective glycoconjugates, with the aim to preserve the immunogenicity of the protein chosen as carrier. The potential protective protein MrkA, the major component of *Klebsiella pneumoniae* type 3 fimbriae, was successfully modified with a lactose moiety in *E. coli*. *K. pneumoniae* K2 K-antigen and O1v1 O-antigen were then covalently linked to MrkA at the level of this unique sugar handle and tested *in vivo*. Immune response against MrkA and sugars was evaluated in animal models. This work contributes to expand the application of the glycoengineering technology for the development of effective glycoconjugate vaccines.

## Abstract 2

In our work we have combined cytoplasmic glycoengineering and chemical conjugation for the development of novel selective glycoconjugates, with the aim to preserve the immunogenicity of the protein chosen as carrier.

## Graphical abstract

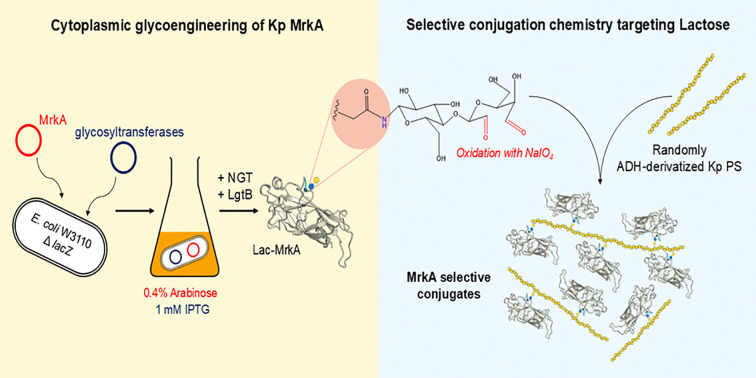

## Introduction

Glycoconjugates are among the safest and most efficacious polysaccharide (PS)-based vaccines developed so far.^1^ The coupling of a PS to an appropriate protein allows to overcome the limitations related to the T-independent nature of PS, providing long-term immunological memory also in infants and elderly.^2^ Currently licensed glycoconjugate vaccines employ the same small set of carrier proteins (diphteria toxoid, tetanus toxoid, CRM_197_, *Haemophilus influenzae* Protein D; and the outer membrane protein complex of meningococcus B),^3^ rising concerns about the pre- or co-exposure to the same carrier that could lead to the reduction of the anti-carbohydrate immune response.^4^ The risk of this so-called carrier-induced epitope suppression phenomenon highlighted the need for new carrier proteins.^3,5^ In this context, interest is growing in exploring the possibility to use a pathogen specific protein simultaneously as carrier and antigen for the generation of “double-hit” vaccines.^3^ As a consequence, selective conjugation chemistries might be necessary to have a limited impact on protein structure and conformation, being mindful of the extent and the location of glycans to be conjugated.^6^ Besides direct chemical modifications strategies,^7–10^ which still have some drawbacks related to selectivity/specificity, and homogeneity of the reactions, genetic engineering has proven to be an invaluable methodology for the controlled introduction of a variety of abiotic and biotic chemical handles at designated sites for subsequent biorthogonal reactions^11^ resulting in the production of homogeneous selective conjugates. In particular, bacterial glycoengineering has emerged as a powerful tool for site-selective protein modification. The discovery that protein glycosylation systems exist also in the bacteria realm^12,13^ paved the way for the development of Protein-Glycan Coupling Technology (PGCT) or bioconjugation, with production of glycoconjugate vaccines directly within bacterial hosts.^14–16^ Recent PGCT-derived glycoconjugate vaccines were developed using a “double-hit” strategy, which could result particularly useful for bacterial species with multiple, diverse glycan structures like pneumococcus.^17^

A more recently discovered glycosylation mechanism, firstly identified in proteobacterium *Haemophilus influenzae*,^18–20^ relies on a *N*-glycosyltransferase (NGT) operating in the bacterial cytoplasm, the cell compartment commonly used for efficient recombinant protein expression. Recently, a biosynthetic pathway for site-specific polysialylation of recombinant proteins in *E. coli* cytoplasm employing NGT for the installation of a priming β-glucose was described.^21^ By the addition of a series of appropriate enzymes (LgtB, SynB and a polysialyltransferase from *N. meningitidis* and CstII from *C. jejuni*), the authors were able to achieve not only polysialylation of a native substrate of NGT, but also of heterologous proteins like super folder Green Fluorescent Protein (sfGFP) or designed ankyrin repeat proteins (DARPin), a class of antibody mimetic with a number of emerging therapeutic application. This technology platform has also been extended to self-assembling proteins for the production of glycosylated megadalton-scale nanoparticles in *E. coli* cytoplasm with diverse future biomedical applications.^22^ The potential of NGT-catalyzed *N*-glycosylation as a tool for the site-specific modification of proteins with glucose sets in fact the basis for many interesting applications.

In our work, we have investigated this technology for the development of novel selective “double-hit” glycoconjugates, with the aim of preserving the antigenicity of the protein chosen as carrier using *Klebsiella pneumoniae* (Kp) as model pathogen. Kp has been listed by WHO among the priority multidrug-resistant pathogens for development of new interventions, being the causative agent of the majority of nosocomial infections and neonatal sepsis in low- and middle- income countries,^23–25^ and no vaccine is yet available.

Epidemiological studies raised the awareness that a Kp vaccine able to cover around 70% of clinically relevant Kp strains should include at least 25 capsular PS, also indicated as K-antigens (KAg).^26^ Among these, K1 and K2 are often found in hypervirulent Kp strains.^27,28^ O-antigens (OAg) are much lower in number compared to KAg and only four of them (O1, O2, O3 and O5) are predicted to cover over 80% of clinically relevant Kp strains.^29–32^ Recently vaccines combining KAg and OAg have been proposed.^33^

Here, both KAg and lipopolysaccharide OAg have been used for the generation of “double-hit” vaccines using MrkA as carrier and potential protective protein antigen. In particular, O1v1 polygalactan and K2 were selected as model OAg and KAg, respectively. MrkA, the major component of Kp type 3 fimbriae, possesses a high degree of sequence conservation among different isolates,^34^ for this reason its use in a glycoconjugate could increase the vaccine coverage, considering the large number of described Kp PS. Moreover, there are already preclinical data showing that immunization with purified fimbriae was able to protect mice against a lethal challenge in a model of acute pneumonia,^35^ and that mice vaccinated subcutaneously with both monomeric and oligomeric MrkA showed a reduction in bacterial burden after intranasal challenge with Kp.^36^

## Results and discussion

### Self-complemented MrkA monomer for cytoplasmic glycoengineering

**Fig. 1:**
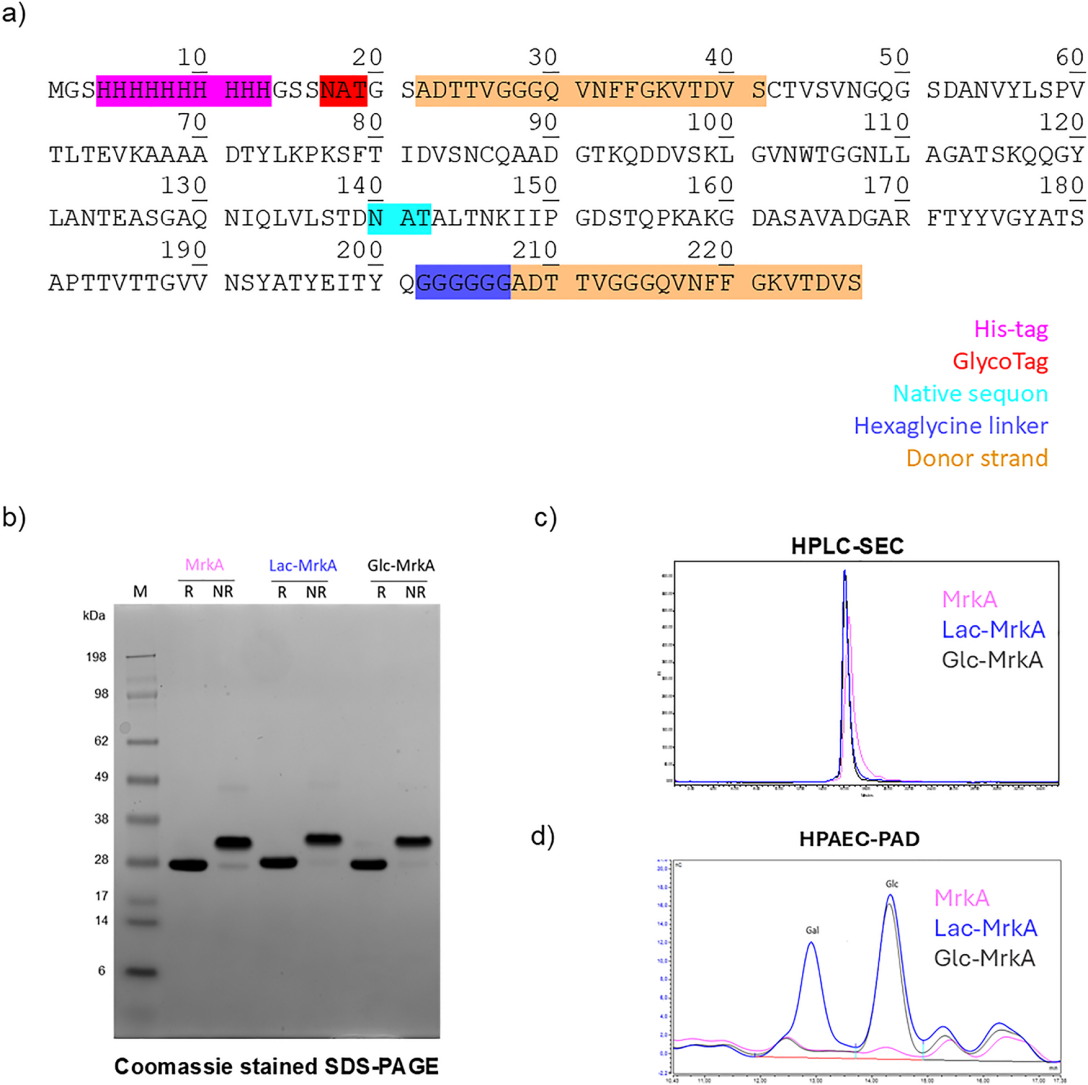
Characterization of purified MrkA and Glyco-MrkA recombinant proteins. (a) Amino acids sequence of self-complemented MrkA monomer. (b) SDS-PAGE analysis of MrkA co-expressed with NGT + LgtB (Lac-MrkA) or with NGT only (Glc-MrkA), compared to the unmodified protein. Due to the presence of a disulfide bond in MrkA, non-reduced (NR) samples bands appear shifted toward higher MW respect to the corresponding ones treated with DTT (R). Lac-MrkA band is also slightly higher in respect to that of MrkA, supporting protein modification evidence. (c) HPLC-SEC overlaid fluorescence emission profiles of MrkA (pink line), Lac-MrkA (blue line) and Glc-MrkA (black line). (d) HPAEC-PAD chromatograms showing Galactose (Gal) presence only in Lac-MrkA (blue line) and Glucose (Glc) presence in Glc-MrkA (black line) and Lac-Mrka, but not in MrkA (pink line). Quantification of Glc in Lac- and Glc-MrkA and Gal in Lac-MrkA was performed in relation to a calibration curve containing a mixture of these neutral sugars, after performing the acid hydrolysis of MrkA samples for the release of the above-mentioned monosaccharides.

MrkA naturally form oligomers whose stability is associated with inter-molecular β sheets in the C-terminal region of the protein^37^ resulting from the interaction with the N-terminal donor strand of each subsequent subunit. To obtain a self-complemented MrkA monomer, the donor strand displacement strategy was used as previously described^38,39^ (Fig. 1a). Expression of recombinant MrkA monomer in *E. coli* under the control of the arabinose-inducible promoter resulted in good yields (∼60 mg L^−1^) of the purified protein (Fig. 1b and c). The biosynthetic pathway for protein glycosylation was established in *E. coli* K12 W3110 strain after deleting *lacZ* gene to prevent lactose catabolism (Fig. S1), confirming previously published results.^22^ This pathway consisted of a first enzyme, ApNGT from *Actinobacillus pleuropneumoniae*, required for protein modification with single β-linked glucose at target asparagine (Asn, N) residue in the NXS/T sequon, where X is not P, recognized by this glycosyltransferase. For generating the N-linked lactose, the β1,4-galactosyltransferase LgtB from *N. meningitidis*,^40^ having a demonstrated substrate promiscuity and active expression in *E. coli*, was used to transfer galactose from the corresponding nucleotide-activated sugar to Glcβ1-Asn handle. These two enzymes were cloned in a compatible vector plasmid, each one under the IPTG-inducible control of LacUV5 promoter, a mutated variant of lac promoter, to allow protein and enzymes co-expression (Fig. S2). This dual plasmid expression system should ensure a good balance between substrate protein and glycosyltransferases synthesis. For our scope, MrkA, with N-A-T sequon inserted at the N-term (Fig. 1a), was co-expressed in the *E. coli* K-12 derivative Δ*lacZ* strain with ApNGT and LgtB enzymes or with ApNGT only. In fact, a truncated pathway construct was produced to follow the sequential MrkA modification and to evaluate which type of sugar handle could better work for the following selective conjugation strategy. As for the unmodified MrkA, Glyco-MrkA recombinant proteins were obtained with a good yield (∼70 mg L^−1^) and purity (Fig. 1b and c). In the protein gel a slight shift of Lac-MrkA band to higher MW can be observed, most likely suggesting modification occurrence. High Performance Anion Exchange Chromatography with Pulsed Amperometric Detection (HPAEC-PAD) was used to quantify the amount of monosaccharides (Glc and Gal) released after acid hydrolysis of the different glycoproteins, to determine the level of glycosylation by calculating the molar ratio of sugar monomers to protein (Fig. 1d). While glucosylation of Glc-MrkA was nearly complete in all analysed lots, lactose occupancy was initially low. Interestingly, the use of a different growth medium, containing dextrose instead of glycerol as carbon source, allowed to significantly increase lactosylation of Lac-MrkA (Table S1).

Successful MrkA glycosylation was additionally confirmed by intact mass analysis (ESI-MS, Fig. S3) and peptide mass fingerprinting following trypsin digestion (LC-MS/MS, Fig. S4). Unexpectedly, the peptide modified by either Glc or Lac identified by LC-MS/MS was not the one specifically added to the N-terminus of MrkA (highlighted in red in Fig. 1a). Instead, the Asn residue of a different NGT sequon naturally present in a solvent-exposed loop of MrkA (highlighted in turquoise in Fig. 1a) resulted to be modified by Glc or Lac in Glc-MrkA or Lac-MrkA, respectively (Fig. S4).

### Exploiting Lac-MrkA for the generation of site-selective double-hit glycoconjugates

Glyco-MrkA was used to develop a selective conjugation approach aimed at preserving MrkA immunogenicity. The unique sugar handle present on the recombinant MrkA monomer has been indeed exploited as a specific target for the site-selective conjugation of Kp PS, both OAg and KAg, *via* reductive amination. Preliminary tests were performed on both MrkA glycoforms to select the best conditions for sugar oxidation, since it directly impacts the conjugation efficiency. At higher NaIO_4_ concentration (5 mM), the oxidation level increased (Table 1). Interestingly, galactose resulted to be more susceptible to oxidation, very likely because of the preferential cleavage of vicinal cis-diols that more easily form the cyclic periodate intermediate with respect to *trans* diols of glucose.^41^ Thus, the lactose glycoform was chosen for the generation of the selective conjugates, since with approximately 50% of sugar oxidation in Glc-MrkA, only half of MrkA molecules would be actually available for the conjugation.

**Table 1:** Oxidation percentages of glucose (in Glc- and Lac-MrkA) and galactose (in Lac-MrkA) in oxidized glyco-proteins determined by anion exchange chromatography

Glyco-protein	2 mM NaIO_4_		5 mM NaIO_4_	
	Glc ox (%)	Gal ox (%)	Glc ox (%)	Gal ox (%)
Glc-MrkA	41	—	58	—
Lac-MrkA	0	83	8	96

**Fig. 2:**
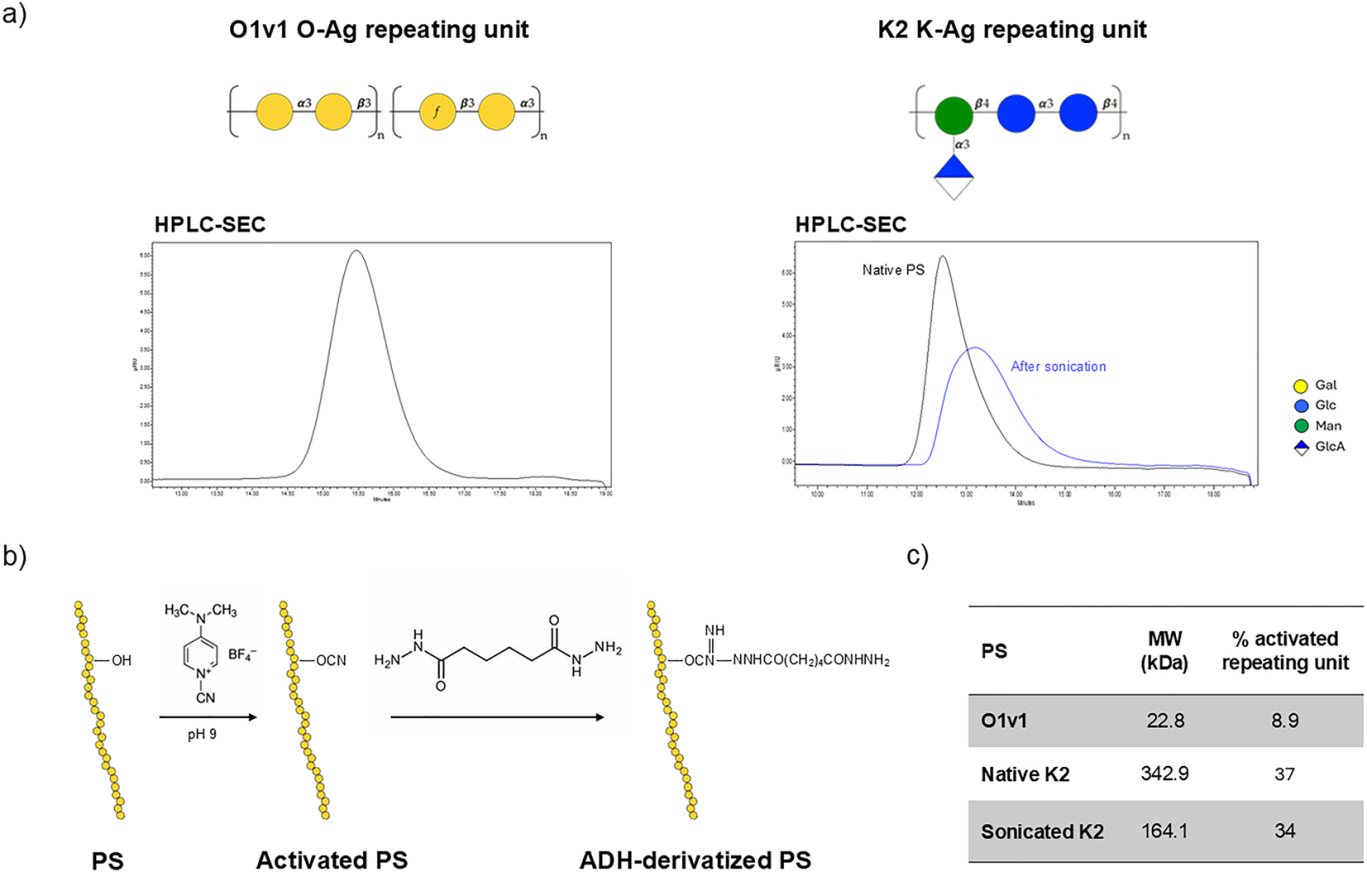
Random activation of O1v1 and K2 with ADH linker through CDAP chemistry. (a) HPLC-SEC profiles of purified O1v1 OAg and K2 KAg (before and after sonication) detected by refractive index. (b) PS hydroxyls groups are activated with CDAP leading to the formation of a cyanoester intermediates for the subsequent reaction with the homobifunctional linker ADH. (c) Characterization of derivatized PS.

Purified O1v1 OAg and K2 KAg (Fig. 2a) were randomly activated with a cyanilating agent (CDAP) and derivatized with the aldehydes-reactive homobifunctional linker ADH, as previously reported^42^ (Fig. 2b). To facilitate protein coupling, the ∼340 kDa K2 was size-reduced to ∼160 kDa by sonication, which decreased solution viscosity (Fig. 2a and c). The activation percentage of size-reduced K2 was comparable to that of the native form (Fig. 2c).

**Fig. 3:**
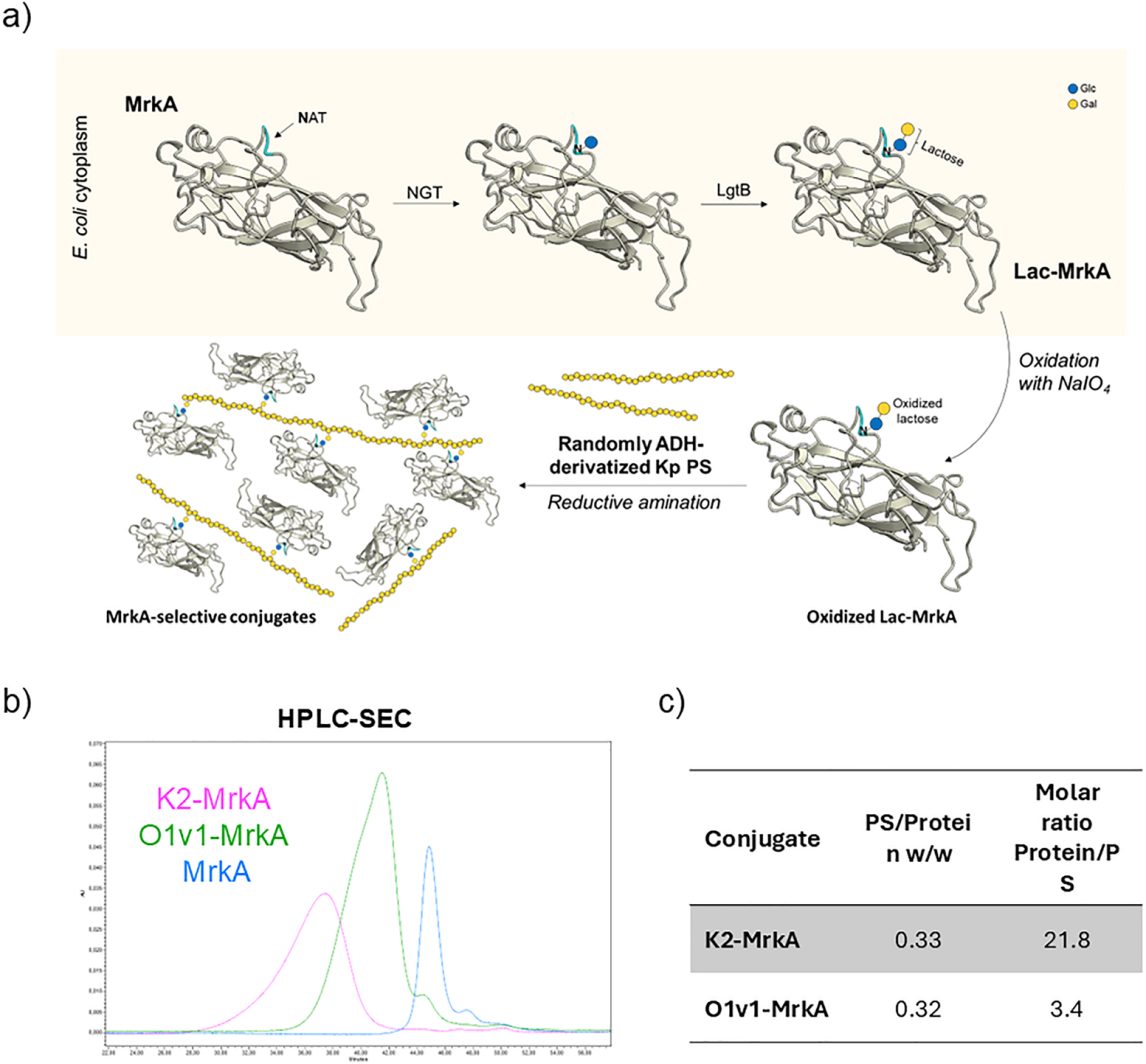
Generation of novel glycoconjugates combining cytoplasmic glycoengineering and selective chemistry. (a) Glycoengineered pathway for site-selective modification of MrkA monomer in *E. coli* cytoplasm followed by a reductive amination reaction scheme leading to site-selective double-hit glycoconjugates. Characterization of purified selective MrkA glycoconjugates: (b) HPLC-SEC profiles in overlay with MrkA recombinant protein and (c) w/w PS/protein and molar protein/PS ratios of each glycoconjugate.

The entire conjugation strategy is schematized in Fig. 3a. Site-selectivity of this reaction was confirmed by SDS-PAGE/WB and HPLC-SEC analyses of a blank reaction between O1v1-ADH and the unmodified MrkA confirming no conjugate formation (Fig. S5).

O1v1 conjugate was entirely recovered in the elution pool of the IMAC purification step, while for K2 conjugate the recovery was lower (70%), probably due to the reduced accessibility of the His-tag to Ni^2+^ ions in a conjugate with a long PS like K2. The lower retention times of the two conjugates, analyzed by HPLC-SEC, with respect to that of MrkA protein, denote an increase in size deriving from the PS conjugation (Fig. 3b). With HPAEC-PAD, combined with microBCA, the molar ratio between MrkA and PS was determined: an average of 3 MrkA molecules was linked to each O1v1 OAg chain, as highlighted also by WB analysis of final purified conjugate (Fig. S6), and of approximately 20 MrkA molecules to K2 KAg (Fig. 3c).

### Immunogenicity of site-selective double-hit glycoconjugates against Kp

**Fig. 4:**
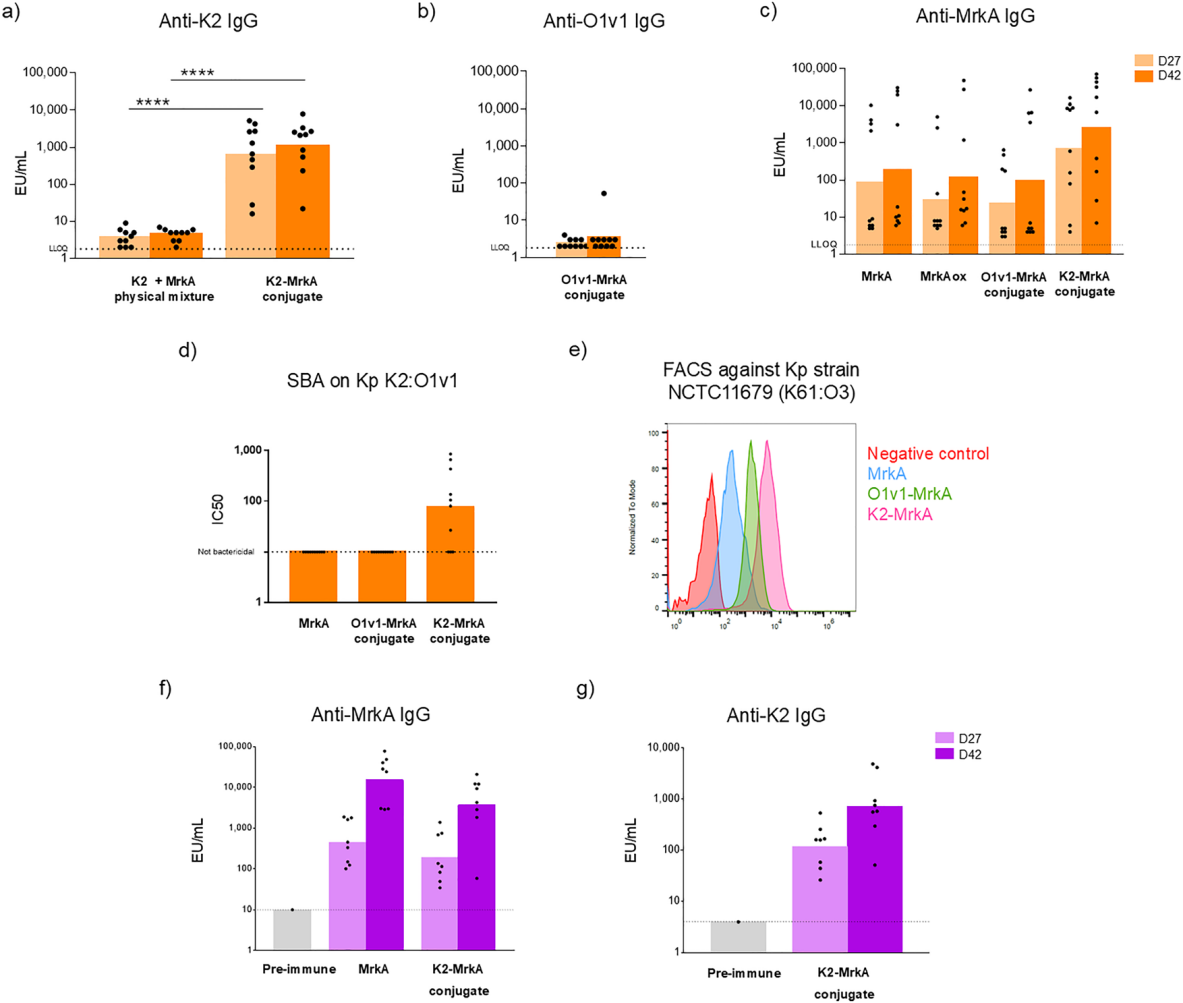
Immunogenicity of MrkA glycoconjugates in mice (orange bars) and rabbits (purple bars). Summary graphs of ELISA results from the mice study reporting individual antibody levels (dots) and geometric mean with 95% confidence interval (bars): anti-K2 specific IgG induced by K2-MrkA conjugate compared to a physical mixture of the two antigens (a), anti-O1v1 specific IgG induced by O1v1-MrkA conjugate (b), and anti-MrkA specific IgG induced by all conjugates tested at 5 µg of protein in comparison to MrkA and Lac-MrkA after NaIO_4_ oxidation (c). SBA graph of D42 sera of mice immunized with MrkA alone and with the two conjugates reporting individual IC50 titers (dots) and geometric mean with 95% confidence interval (bars) against K2:O1v1 Kp strain NCTC11228 (d). Flow cytometry histograms of a heterologous Kp strain (K61:O3) incubated with Day 42 pooled mice sera (1 : 500 dilution) of MrkA alone, O1v1-MrkA, K2-MrkA and PBS 1× as negative control (e). Summary graphs of ELISA results from the rabbit study reporting individual antibody levels (dots) and geometric mean with 95% confidence interval (bars): anti-MrkA specific IgG induced by K2-MrkA conjugate compared to protein alone (f) and anti-K2 specific IgG induced by K2-MrkA conjugate (g).

O1v1-MrkA and K2-MrkA conjugates were tested in mice to investigate the ability of MrkA to work as carrier for Kp PS and as antigen and evaluate the impact of conjugation on MrkA immune response. Mice were immunized subcutaneously twice (at day 0 and 28) at the same MrkA dose of 5 µg in presence of Alhydrogel. To verify if the oxidation step required for the selective conjugation reaction could affect MrkA immunogenicity, Lac-MrkA was oxidized and tested in animals too. As negative control, a physical mixture of K2 with MrkA was included in the immunization scheme. When conjugated to MrkA, K2 induced a significantly higher level of anti-K2 specific IgG titers compared to the physical mixture of the two antigens (*p* < 0.0001 at both day 27 and 42, Fig. 4a). O1v1-MrkA conjugate was instead not able to elicit any anti-O1v1 specific IgG antibodies (Fig. 4b). This could be due to the small size of this conjugate, composed by an average of 3 MrkA molecules selectively linked to the shorter O1v1 sugar, that may not be the most appropriate for immune stimulation. Sugar length and size and macromolecular/multimolecular structure of the conjugate may play indeed a significant role in the production of a good anti-PS IgG response.^43^ Oxidation of Lac-MrkA did not have any impact on the anti-protein immune response, as no difference was observed by comparing MrkA and oxidized Lac-MrkA IgG responses at both day 27 and 42 (Fig. 4c). The anti-MrkA IgG response induced by the two selective glycoconjugates was not significantly different from that of MrkA alone, confirming that conjugation did not affect MrkA immunogenicity (Fig. 4c). To be noted that a high number of non-responders was present in all the groups except for the K2-MrkA conjugate group. At day 42, the GeoMean of the K2-MrkA conjugate group was 14-fold higher than that of MrkA group. It seemed that anti-MrkA IgG response can be improved by the presentation of multiple copies (∼20) of the protein along the PS chain. Interestingly, Serum Bactericidal Activity (SBA) assay against a Kp K2:O1v1 strain expressing MrkA showed ability of K2-MrkA antisera only to kill bacteria (Fig. 4d). In order to verify the ability of anti-MrkA antibodies to bind bacteria, mice sera were also tested by FACS for their ability to bind MrkA expressed as fimbriae (and not recombinant monomer) on a heterologous Kp strain selected for high expression of type 3 fimbriae and mismatched KAg and OAg. Interestingly, antibodies were not only able to recognize the recombinant protein monomer in ELISA, but also the natural MrkA oligomer displayed on bacteria surface. In agreement with anti-MrkA ELISA results, sera from mice immunized with K2-MrkA conjugate showed a higher binding ability compared to those of O1v1-MrkA conjugate and protein alone (Fig. 4e).

K2-MrkA conjugate was also tested in rabbits. Also in this animal model, where the glycoconjugate and MrkA alone were compared at the same protein dose of 15 µg, in presence of Alhydrogel, K2-MrkA conjugate elicited a good level of anti-MrkA IgG that was not significantly different from that induced by MrkA alone (Fig. 4f). Therefore, the ability of MrkA to work as carrier and pathogen specific antigen when linked to K2 was confirmed also in a different animal species (Fig. 4g).

### Further explorations of cytoplasmic glycoengineering

**Fig. 5:**
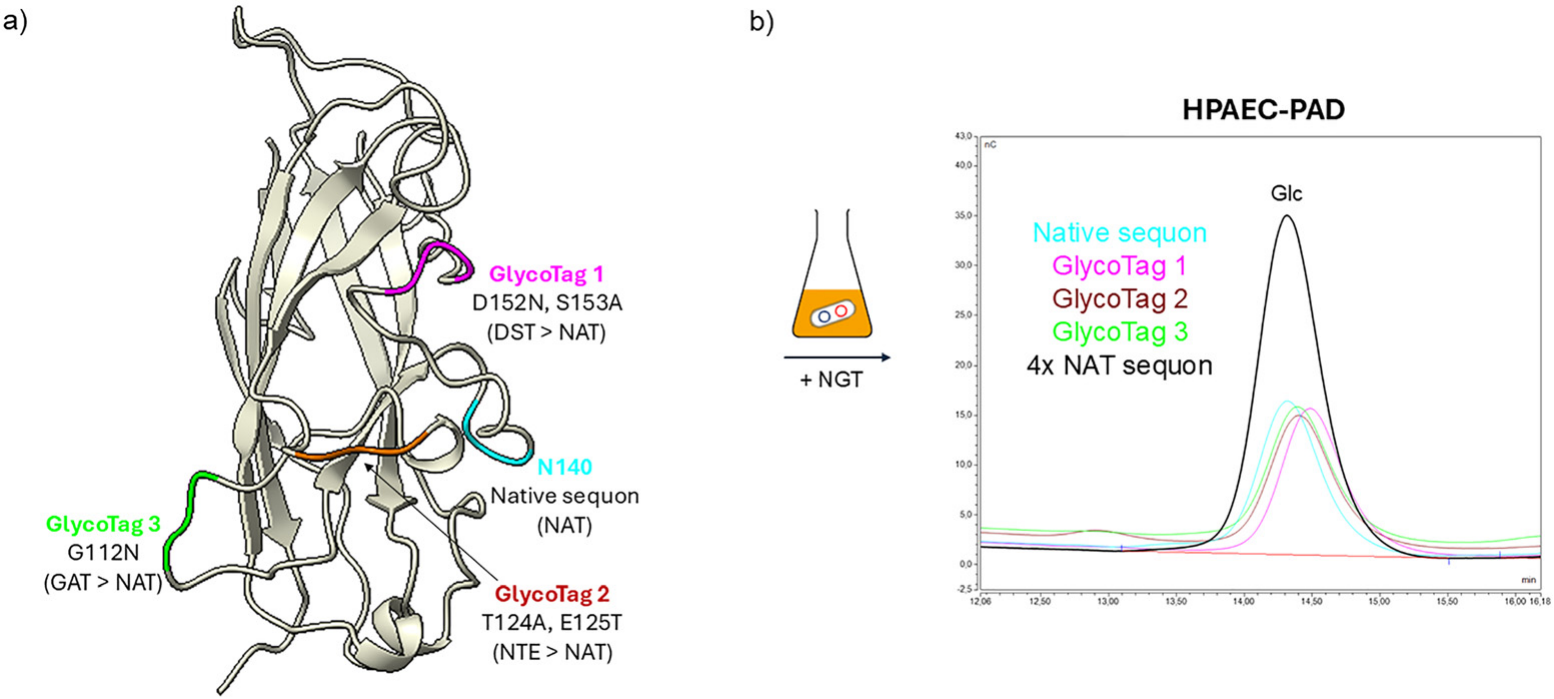
Testing the boundaries of cytoplasmic glycoengineering. (a) Self-complemented MrkA structure (PDB ID 9HW9) showing the position of each sequon, native (in turquoise) or engineered, in protein exposed loops. (b) HPAEC-PAD chromatograms showing the amount of glucose (Glc) in the different MrkA versions.

Having discovered through LC-MS/MS that Lac modification occurred at the level of a sequon naturally occurring in MrkA amino acid sequence and not on the one added at the N-term of the protein sequence, different MrkA versions were designed to better characterize the activity of the glycosyltransferases. This allows to explore if conjugation site can have an impact on conjugate immunogenicity and on the antigenicity of the protein. To reduce the complexity of the system, only NGT activity was studied, knowing that glucose modification was complete and consistent (Table S1). Three additional MrkA constructs were designed and produced, mutating the glycosylated Asn into Gly in the native sequon, and engineering NGT sequons as GlycoTags in different positions of MrkA sequence. Peptide mass fingerprint analysis by LC-MS/MS was performed in order to get information on the modification site. The data showed that the residue involved in glycosylation was the expected Asn for each costruct (Fig. S4). To be noted that only when we located the sequon at the N terminus of MrkA, the expected modification did not take place. This could be probably due to the intrinsically disordered nature of this region. Moreover, it is reported that *N*-glycosylation occurs with less probability in the termini of protein sequences compared to the mid region of *N*-glycoproteins.^44,45^ In agreement with LC-MS/MS, HPAEC-PAD analysis confirmed 100% of Glc modification in all the three constructs, compared to the previously described Glc-MrkA (Fig. 5b). Finally, a fifth MrkA version was also designed carrying up to four NGT sequons. HPAEC-PAD analysis showed a molar ratio of Glc to protein of ∼4, meaning that modification of each sequon was close to 100% (Fig. 5b).

## Conclusion

In this work, we developed a novel selective conjugation strategy based on cytoplasmic protein glycoengineering, testing Kp type 3 fimbriae MrkA as protein carrier and antigen. MrkA has gained strong interest as a potential vaccine target, being expressed by most of Kp strains, including hv-Kp. It is present with a sequence homology of above 85% in approximately 95% of Kp isolates from the Institute Pasteur database (∼40 000 genomes), therefore a double-hit approach, meaning that both PS and carrier protein belong to the same pathogen, may be very useful to increase vaccine coverage for a bacterial species with large glycan variability like Kp.^46^

Cytoplasmic glycoengineering was successfully applied for the modification of MrkA with glucose and lactose. The lactose glycoform was better oxidized with respect to Glc-MrkA, so oxidized Lac-MrkA was used for the reductive amination reaction with the randomly ADH-derivatized PS. This selective conjugation reaction led to the formation of glycoconjugates characterized by several protein molecules linked through their single attachment point to the PS chains, differently from bioconjugates where one protein carries few glycan chains. Overall, it appears that the ability of MrkA to work as a carrier for PS is proportional to the molecular mass of the final glycoconjugate. Indeed, the K2-MrkA conjugate was able to induce a good level of anti-PS IgG antibodies, that were also bactericidal against a Kp K2:O1v1 strain, contrary to the OAg conjugate. Importantly selective conjugation of MrkA to PS did not negatively impact the immune response elicited by the protein. Actually, conjugation of many proteins to long K2 chains resulted in a slightly improved IgG response against MrkA, confirmed by testing the binding ability of anti-MrkA antibodies induced to a wild type Kp strain with mismatched OAg and KAg *via* flow cytometry.

The main limitation of this work is that MrkA functional epitopes are still unknown, therefore we do not know where modification of MrkA could be more appropriate not to impact sera functionality. While established functional assay to measure the potency of anti-MrkA monoclonal antibodies have been described, including the biofilm inhibition assay and the opsono-phagocytic killing (OPK) assay, their application to animal sera remains challenging, making it hard to determine the real impact of glycosylation on MrkA antigenicity. Nevertheless, we have demonstrated that NGT sequons can be easily engineered in different sites of the protein supporting additional studies to understand if the glycosylation position can affect the immunogenicity of resulting selective conjugates. Given the results from the mice study with the O1v1-MrkA conjugate, more than one sequon could be necessary to induce good anti-PS immune responses for a shorter PS like this one. The low immunogenicity of the O1v1-MrkA conjugate could be related to the poor carrier function of a small protein antigen as MrkA attached to a short sugar chain through one single attachment point. Interestingly, same results have been obtained with MAPS technology that resembles the same final saccharide-protein presentation.^39^ Very likely a higher glycan loading on the protein could be needed for short oligosaccharides to result in an optimal immune response.^43^ Indeed, other approaches reported in literature allowed successful induction of significant anti-OAg IgG responses.^33,47–50^ In conclusion, we successfully combined bacterial cytoplasmic glycoengineering and selective conjugation chemistry for the generation of a Kp double-hit vaccine. The approach developed here can be extended to other pathogens for the synthesis of innovative glycoconjugates selectively linking long chain PS to proteins, the latter having the double role of carrier and antigen.

## Author contributions

FM and GG conceived the study; EP, VM and SD performed experimental work; EP, FM and GG analyzed the results; EP, FM and GG wrote the manuscript; all revised the manuscript.

## Conflicts of interest

This work was sponsored by GlaxoSmithKline Biologicals SA. GSK Vaccines Institute for Global Health is an affiliate of GlaxoSmithKline Biologicals SA. Elena Palmieri, Francesca Micoli and Gianmarco Gasperini are employed by the GSK group of companies. Elena Palmieri was a student at the University of Trieste and participated in a post graduate studentship program at GSK at the time of the study. Valentina Monaci was a student at the University of Florence and participated in a post graduate studentship program at GSK at the time of the study.

## Supplementary Material

CB-007-D5CB00320B-s001

## Data Availability

The data supporting this work has been included as part of the article and the supplementary information (SI). Supplementary information is available. See DOI: https://doi.org/10.1039/d5cb00320b.
