# Supplementary material for: Double-hit approach for novel glycoconjugates combining cytoplasmic glycoengineering and selective chemistry
Source: RSC Chem Biol. 2026 Jan 15;7(3):464–72. doi: 10.1039/d5cb00320b (PMC12805384; doi:10.1039/d5cb00320b)
Supplement: CB-007-D5CB00320B-s001 [file CB-007-D5CB00320B-s001.pdf]

Supplementary Information (SI) for RSC Chemical Biology.
This journal is © The Royal Society of Chemistry 2026

Supplementary information

Double-hit approach for novel glycoconjugates combining cytoplasmic glycoengineering and selective chemistry

Elena Palmieri [1,4], Valentina Monaci [1,2], Salvatore Durante [3], Paola Cescutti [4], Francesca Micoli[1], Gianmarco Gasperini [3*]

[1]*GSK Vaccines Institute for Global Health (GVGH), Via Fiorentina 1, 53100 Siena, Italy*

[2]*Resonance Center - CERM, University of Florence, Via Luigi Sacconi 6, Sesto Fiorentino, 50019, Florence, Italy.*

[3]*GSK Vaccines Srl, Via Fiorentina 1, 53100 Siena, Italy*

[4]*Department of Life Sciences, University of Trieste, Via L. Giorgieri 1, Bldg C11, 34127 Trieste, Italy*

[*]Corresponding author: gianmarco.x.gasperini@gsk.com

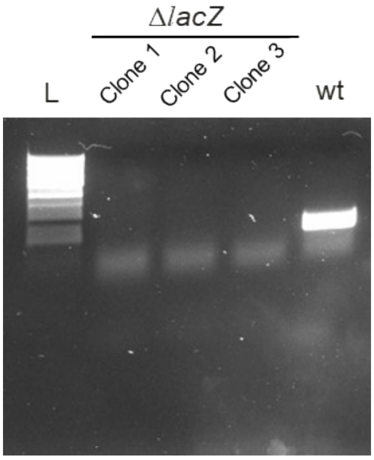

Figure S1. Agarose gel of PCR amplicons showing the absence of lacZ gene in the three clones of the mutated *E. coli* K12 W3110 strain and its presence instead in the wild-type positive control strain.

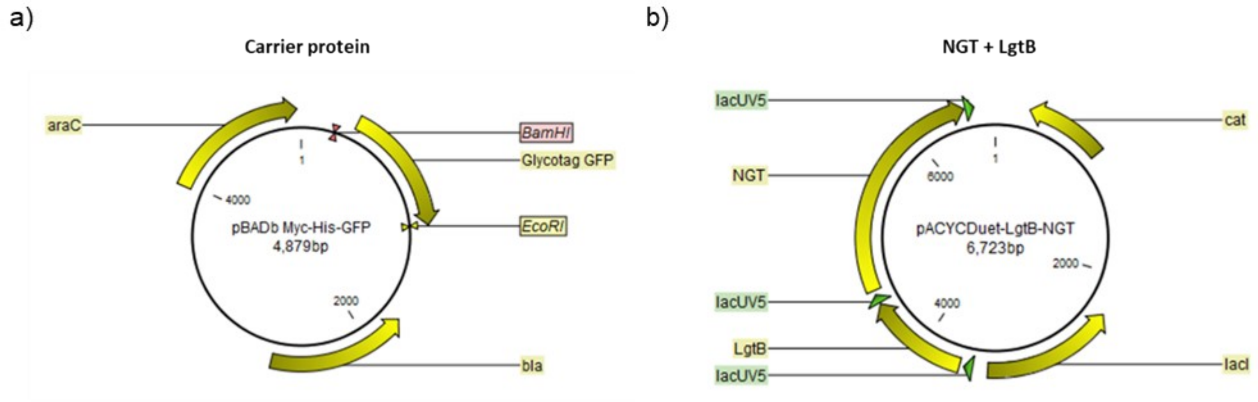

Figure S2. Plasmid maps of vectors used for expression of sfGFP (or carrier protein) a) and glycosyltransferases b).

Table S1. Molar percentage of lactose or glucose in MrkA recombinant protein co-expressed with NGT and LgtB in *E. coli* W3110 Δ*lacZ* grown in HTMC or modified HTMC (containing 5 g/L of dextrose instead of 15 g/L of glycerol as carbon source). Modified HTMC allowed to improve efficiency of modification and reduce batch-to-batch variability.

**Batch/Growth medium**	***Molar ratio Lac*/protein (%)***	***Molar ratio Glc/protein (%)***
**Batch 1/HTMC**	***6***	***96***
**Batch 2/HTMC**	***21***	***100***
**Batch 1/Modified HTMC**	***70***	***100***
**Batch 2/Modified HTMC**	***100***	***100***
**Batch 3/Modified HTMC**	***82***	***98***

*Lac amount calculated based on amount of Gal by HPAEC-PAD analysis

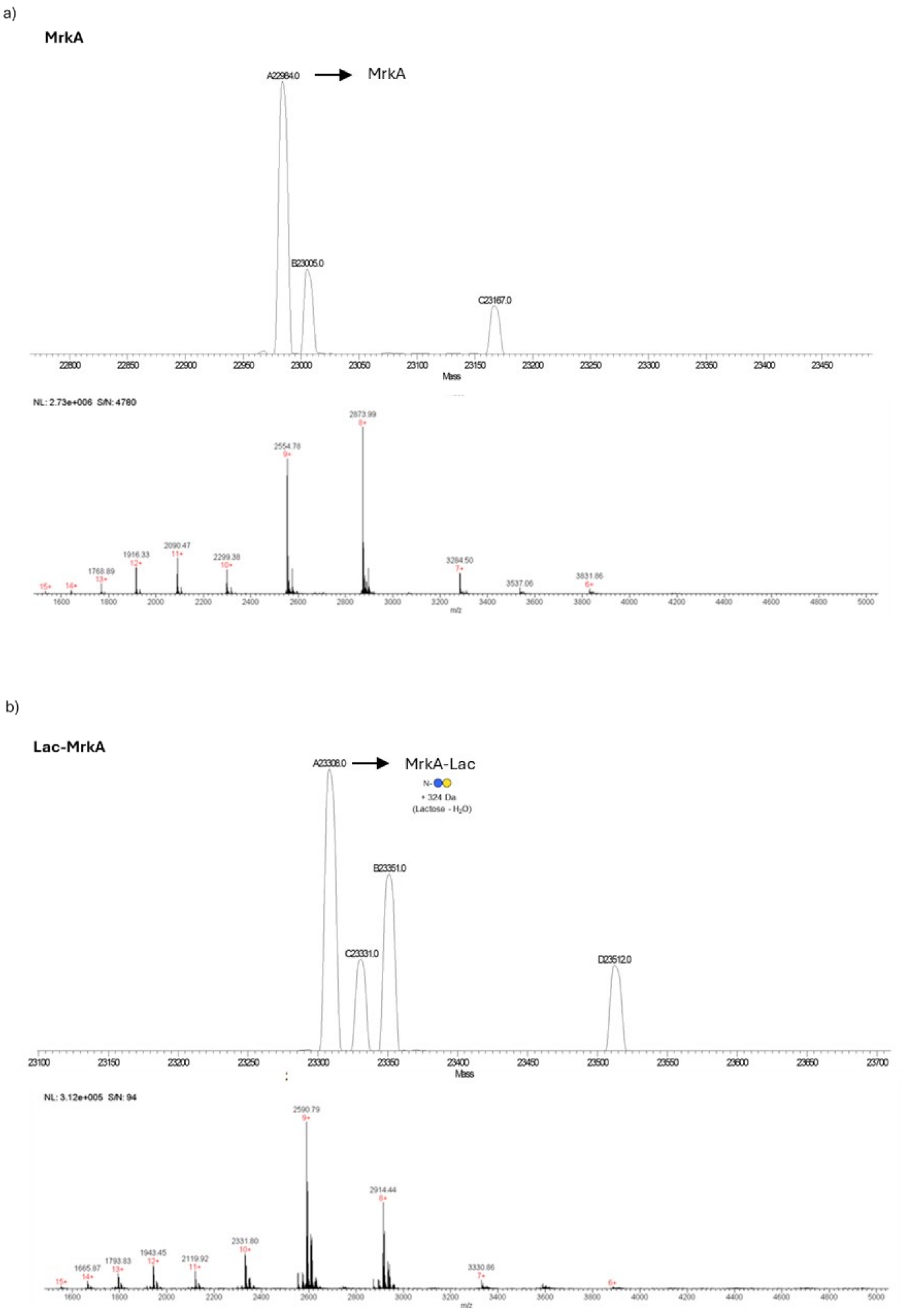

Figure S3. Deconvoluted (top) and raw (bottom) ESI-MS spectra of unmodified MrkA (a) and Lac-MrkA (b)

a)

MrkA

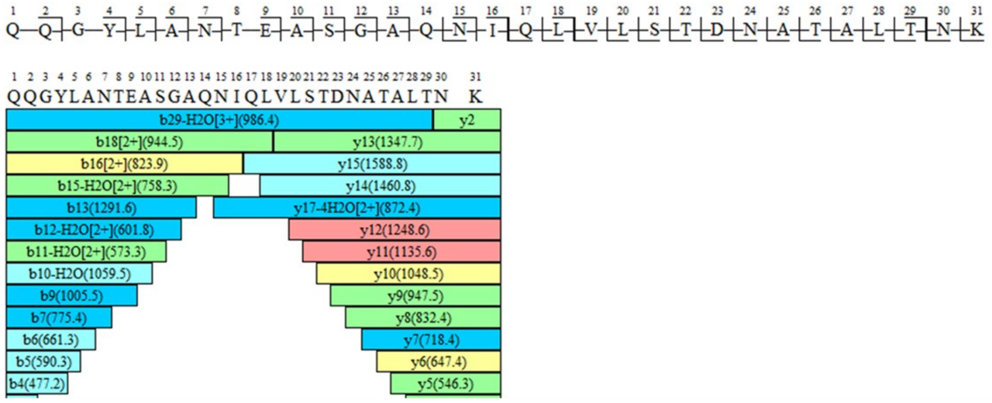

Q Q G Y L A N T E A S G A Q N I Q L V L S T D N A T A L T N K
b29-H2O[3+](986.4)
y2
b18[2+](944.5)
y13(1347.7)
b16[2+](823.9)
y15(1588.8)
b15-H2O[2+](758.3)
y14(1460.8)
b13(1291.6)
y17-4H2O[2+](872.4)
b12-H2O[2+](601.8)
y12(1248.6)
b11-H2O[2+](573.3)
y11(1135.6)
b10-H2O(1059.5)
y10(1048.5)
b9(1005.5)
y9(947.5)
b7(775.4)
y8(832.4)
b6(661.3)
y7(718.4)
b5(590.3)
y6(647.4)
b4(477.2)
y5(546.3)

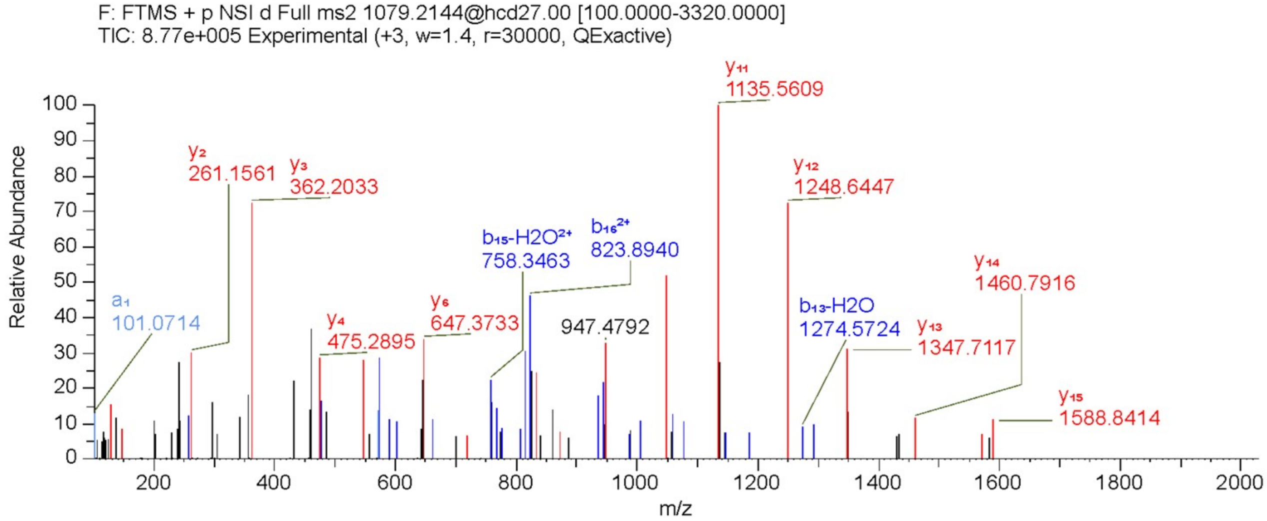

F: FTMS + p NSI d Full ms2 1079.2144@hcd27.00 [100.0000-3320.0000]
TIC: 8.77e+005 Experimental (+3, w=1.4, r=30000, QExactive)
Relative Abundance
m/z
a1 101.0714
y2 261.1561
y3 362.2033
y4 475.2895
y6 647.3733
b15-H2O2+ 758.3463
b16 2+ 823.8940
947.4792
y11 1135.5609
y12 1248.6447
b13-H2O 1274.5724
y13 1347.7117
y14 1460.7916
y15 1588.8414

b)

Lac-MrkA

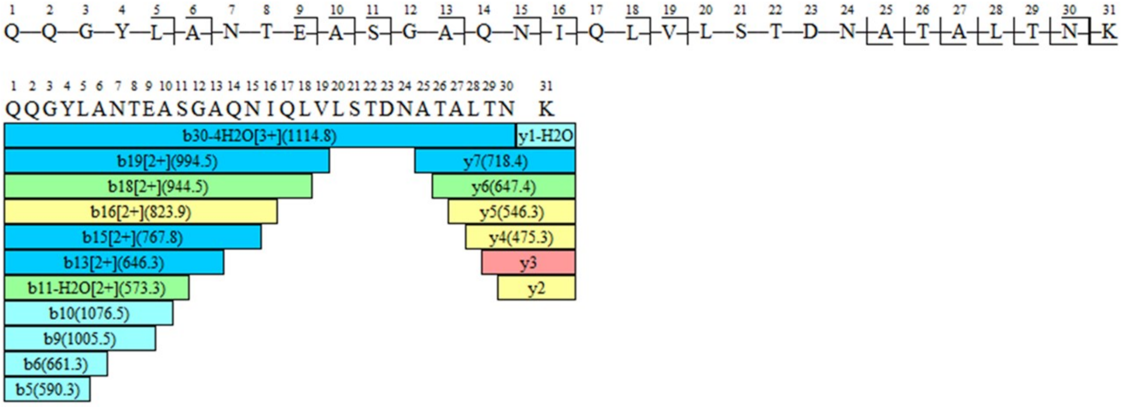

Q Q G Y L A N T E A S G A Q N I Q L V L S T D N A T A L T N K
b30-4H2O[3+](1114.8)
y1-H2O
b19[2+](994.5)
y7(718.4)
b18[2+](944.5)
y6(647.4)
b16[2+](823.9)
y5(546.3)
b15[2+](767.8)
y4(475.3)
b13[2+](646.3)
y3
b11-H2O[2+](573.3)
y2
b10(1076.5)
b9(1005.5)
b6(661.3)
b5(590.3)

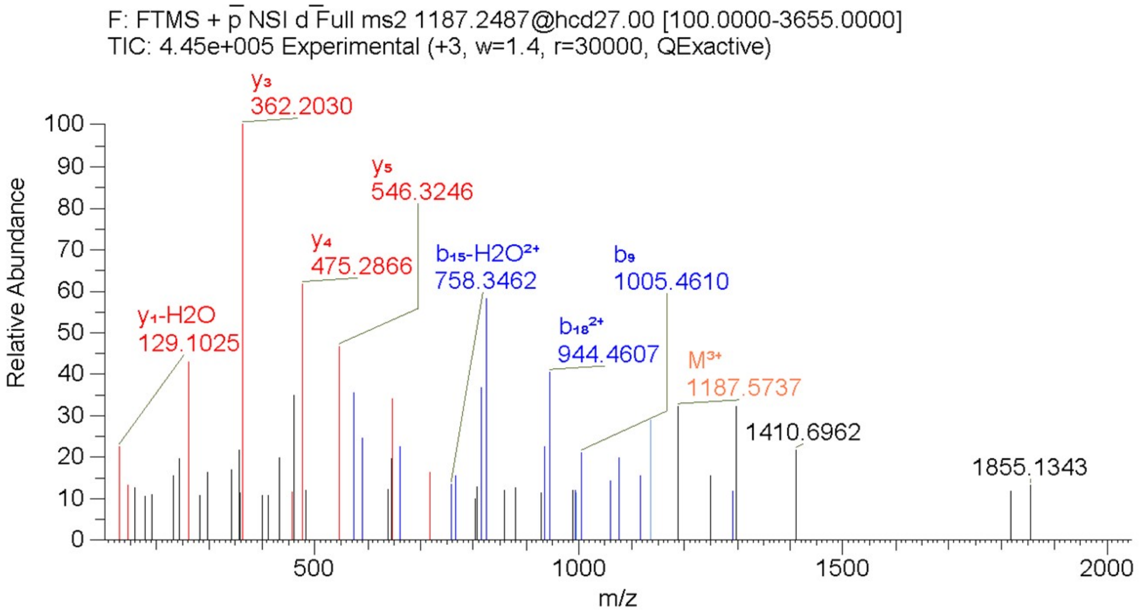

F: FTMS + p NSI d Full ms2 1187.2487@hcd27.00 [100.0000-3655.0000]
TIC: 4.45e+005 Experimental (+3, w=1.4, r=30000, QExactive)
Relative Abundance
m/z
y3 362.2030
y5 546.3246
y4 475.2866
b15-H2O2+ 758.3462
b9 1005.4610
y1-H2O 129.1025
b18 2+ 944.4607
M3+ 1187.5737
1410.6962
1855.1343

c)

Glc-MrkA

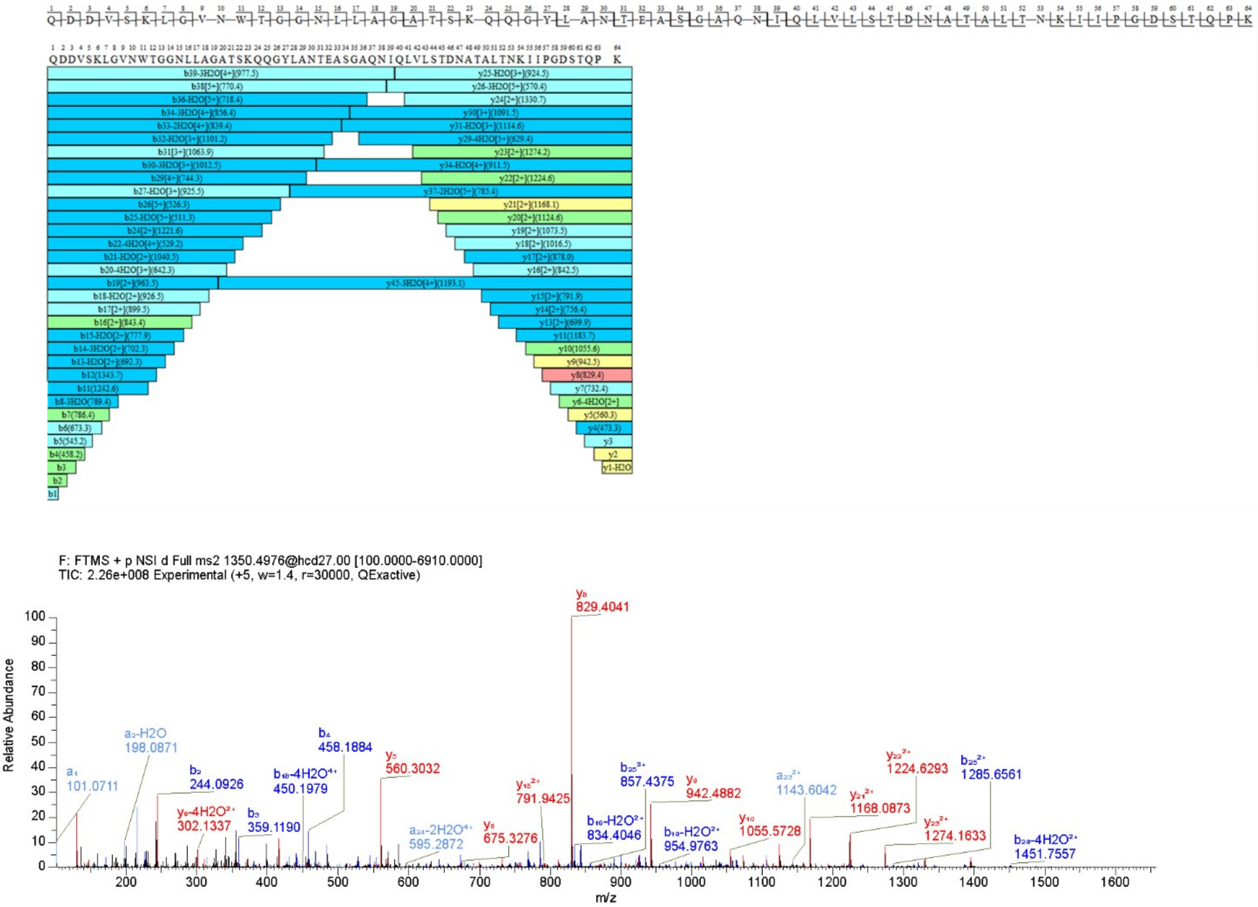
QDDVSKLGVNWTGGNLLAGATSKQQGYLANTEASGAQNIQLVLSTDNATALTNKIIPGDSTQP K
F: FTMS + p NSI d Full ms2 1350.4976@hcd27.00 [100.0000-6910.0000]
TIC: 2.26e+008 Experimental (+5, w=1.4, r=30000, QExactive)
Relative Abundance
m/z
y8 829.4041
a1 101.0711
a2-H2O 198.0871
b2 244.0926
y6-4H2O2+ 302.1337
b3 359.1190
b16-4H2O4+ 450.1979
b4 458.1884
y5 560.3032
a24-2H2O4+ 595.2872
y6 675.3276
y15 2+ 791.9425
b16-H2O2+ 834.4046
b25 3+ 857.4375
y9 942.4882
b19-H2O2+ 954.9763
y10 1055.5728
a23 2+ 1143.6042
y21 2+ 1168.0873
y22 2+ 1224.6293
y23 2+ 1274.1633
b25 2+ 1285.6561
b26-4H2O2+ 1451.7557

d)

GlycoTag 1

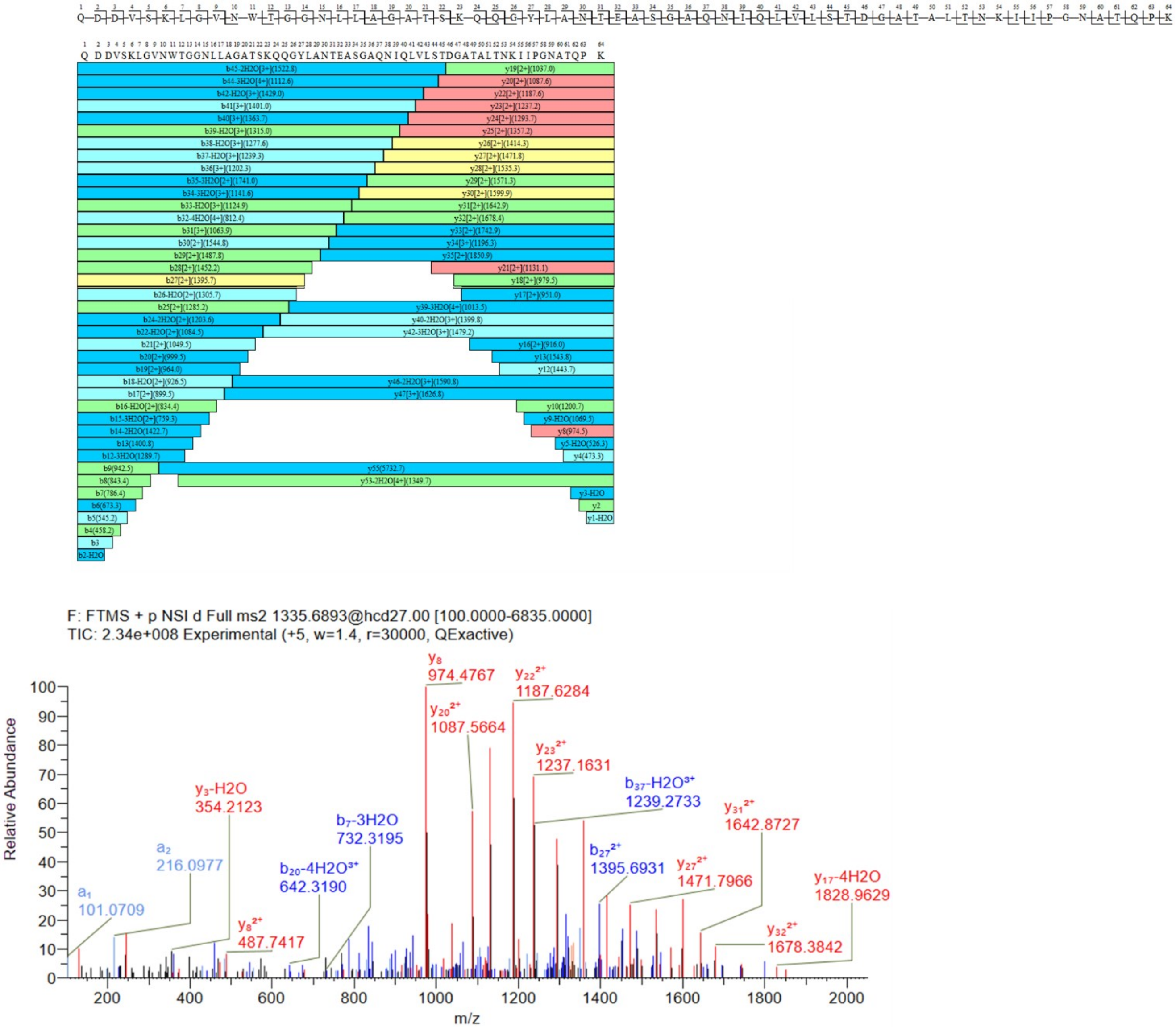
F: FTMS + p NSI d Full ms2 1335.6893@hcd27.00 [100.0000-6835.0000]
TIC: 2.34e+008 Experimental (+5, w=1.4, r=30000, QExactive)
Relative Abundance
m/z
a1 101.0709
a2 216.0977
y3-H2O 354.2123
y8 2+ 487.7417
b20-4H2O 3+ 642.3190
b7-3H2O 732.3195
y8 974.4767
y20 2+ 1087.5664
y22 2+ 1187.6284
y23 2+ 1237.1631
b37-H2O 3+ 1239.2733
b27 2+ 1395.6931
y27 2+ 1471.7966
y31 2+ 1642.8727
y32 2+ 1678.3842
y17-4H2O 1828.9629

e)

GlycoTag 2

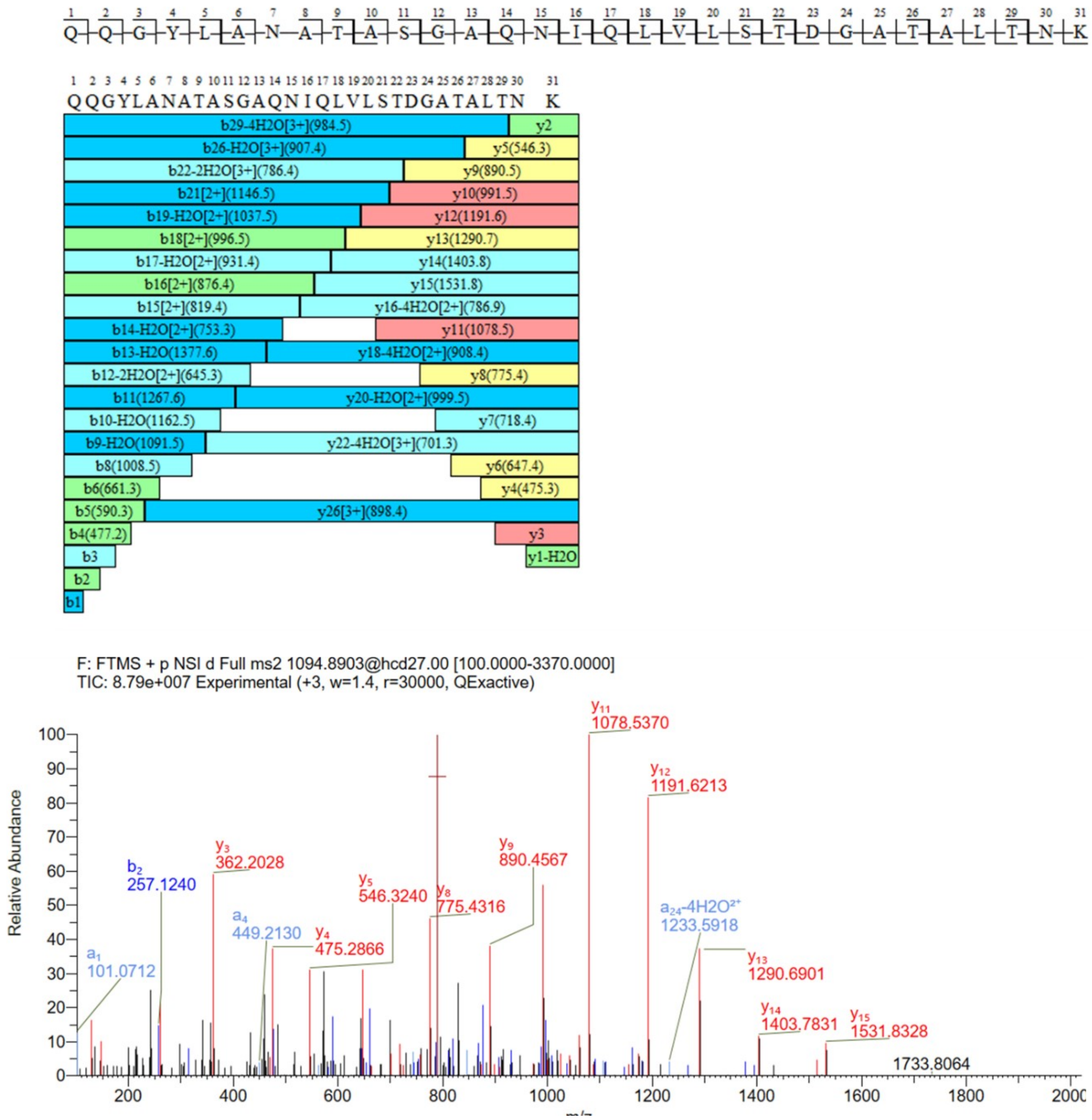
Q Q G Y L A N A T A S G A Q N I Q L V L S T D G A T A L T N K
b29-4H2O[3+](984.5)
y2
b26-H2O[3+](907.4)
y5(546.3)
b22-2H2O[3+](786.4)
y9(890.5)
b21[2+](1146.5)
y10(991.5)
b19-H2O[2+](1037.5)
y12(1191.6)
b18[2+](996.5)
y13(1290.7)
b17-H2O[2+](931.4)
y14(1403.8)
b16[2+](876.4)
y15(1531.8)
b15[2+](819.4)
y16-4H2O[2+](786.9)
b14-H2O[2+](753.3)
y11(1078.5)
b13-H2O(1377.6)
y18-4H2O[2+](908.4)
b12-2H2O[2+](645.3)
y8(775.4)
b11(1267.6)
y20-H2O[2+](999.5)
b10-H2O(1162.5)
y7(718.4)
b9-H2O(1091.5)
y22-4H2O[3+](701.3)
b8(1008.5)
y6(647.4)
b6(661.3)
y4(475.3)
b5(590.3)
y26[3+](898.4)
b4(477.2)
y3
b3
y1-H2O
b2
b1
F: FTMS + p NSI d Full ms2 1094.8903@hcd27.00 [100.0000-3370.0000]
TIC: 8.79e+007 Experimental (+3, w=1.4, r=30000, QExactive)
Relative Abundance
a1 101.0712
b2 257.1240
y3 362.2028
a4 449.2130
y4 475.2866
y5 546.3240
y8 775.4316
y9 890.4567
y11 1078.5370
y12 1191.6213
a24-4H2O2+ 1233.5918
y13 1290.6901
y14 1403.7831
y15 1531.8328
1733.8064

f)

GlycoTag 3

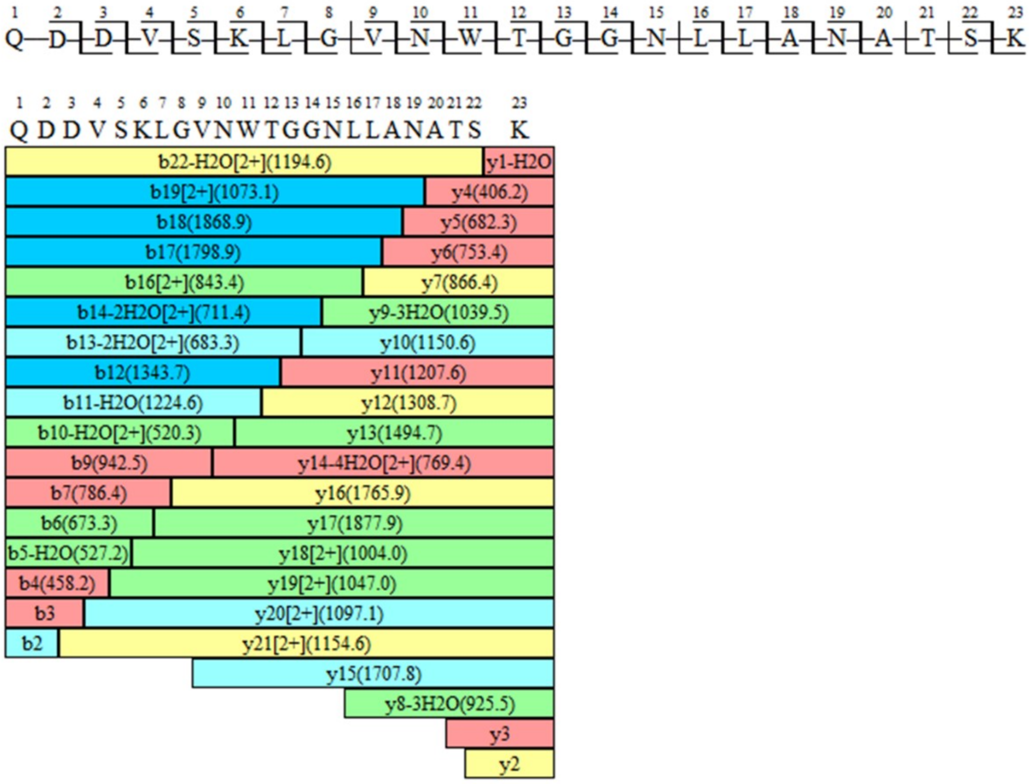

Q D D V S K L G V N W T G G N L L A N A T S K
b22-H2O[2+](1194.6)
y1-H2O
b19[2+](1073.1)
y4(406.2)
b18(1868.9)
y5(682.3)
b17(1798.9)
y6(753.4)
b16[2+](843.4)
y7(866.4)
b14-2H2O[2+](711.4)
y9-3H2O(1039.5)
b13-2H2O[2+](683.3)
y10(1150.6)
b12(1343.7)
y11(1207.6)
b11-H2O(1224.6)
y12(1308.7)
b10-H2O[2+](520.3)
y13(1494.7)
b9(942.5)
y14-4H2O[2+](769.4)
b7(786.4)
y16(1765.9)
b6(673.3)
y17(1877.9)
b5-H2O(527.2)
y18[2+](1004.0)
b4(458.2)
y19[2+](1047.0)
b3
y20[2+](1097.1)
b2
y21[2+](1154.6)
y15(1707.8)
y8-3H2O(925.5)
y3
y2

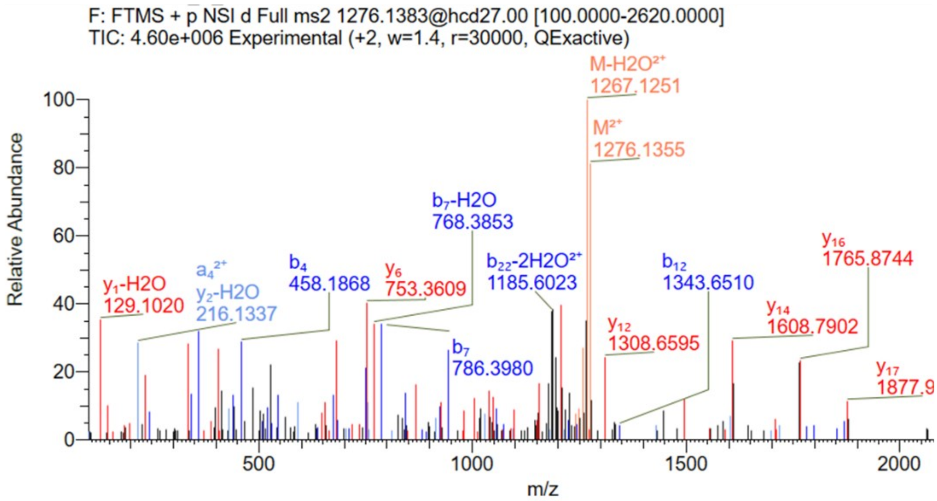

F: FTMS + p NSI d Full ms2 1276.1383@hcd27.00 [100.0000-2620.0000]
TIC: 4.60e+006 Experimental (+2, w=1.4, r=30000, QExactive)
M-H2O2+ 1267.1251
M2+ 1276.1355
b7-H2O 768.3853
b22-2H2O2+ 1185.6023
b12 1343.6510
y16 1765.8744
y1-H2O 129.1020
a4 2+ y2-H2O 216.1337
b4 458.1868
y6 753.3609
y12 1308.6595
y14 1608.7902
b7 786.3980
y17 1877.9
Relative Abundance
m/z

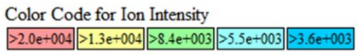

Color Code for Ion Intensity
>2.0e+004
>1.3e+004
>8.4e+003
>5.5e+003
>3.6e+003

Figure S4. Graphical representation (top) and corresponding annotated MS/MS fragmentation spectrum (bottom) of modified peptides identified by LC-MS/MS for unmodified MrkA (a), Glc-MrkA (b), Lac-MrkA (c), GlycoTag 1 (d), GlycoTag 2 (e), GlycoTag 3 (f). The color code of the graphical representation reflects the ionic intensity.

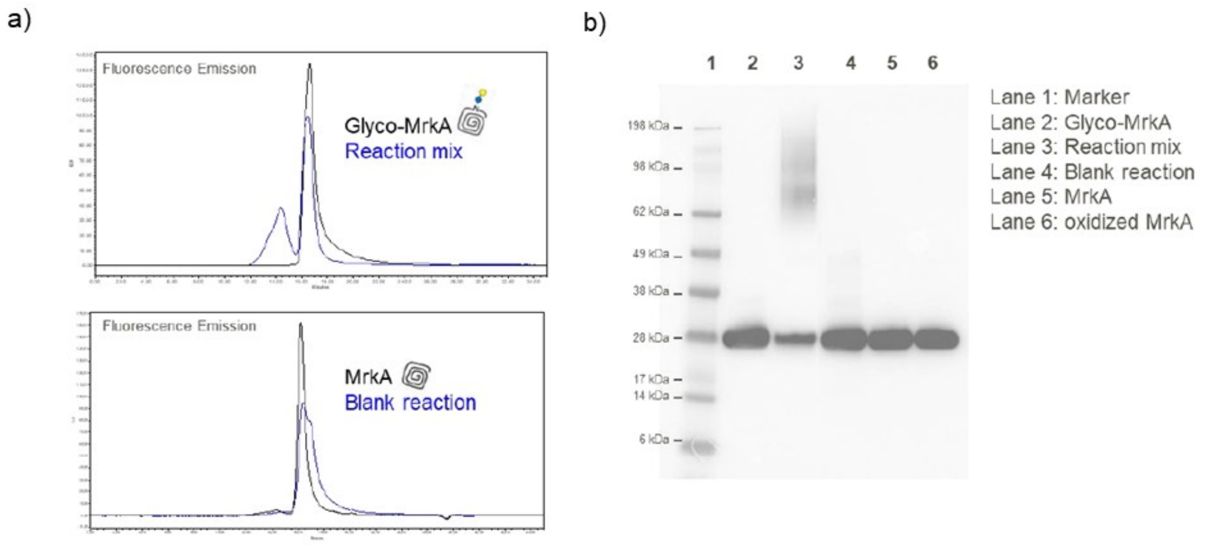

Figure S5. HPLC-SEC and SDS-PAGE/WB analyses confirming the selectivity of the reaction: a) absence of a peak at conjugate retention time (RT) in the Blank reaction chromatogram (second plot) and b) no smear visible at higher MW in the anti-MrkA WB (lane 4 vs. lane 3).

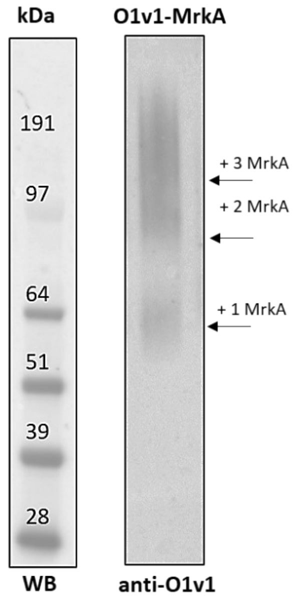

Figure S6. Western Blot analysis of showing a MW ladder reflecting the number of MrkA monomers covalently attached along the OAg chain in the purified O1v1-MrkA conjugate.

Materials and Methods

Bacterial strains, mutants generation and growth/expression conditions

Kp strains NCTC11228 (K2:O1v1) and NCTC11679 (K61:O3) were obtained from Public Health England. NCTC11228 strain was mutated to delete the *tolR* gene for Generalized Modules for Membrane Antigens (GMMA) production. *E. coli* K12 W3110 was acquired from ATCC (27325). *E. coli* K12 W3110 was selected for the generation of *lacZ* mutant, to be used as expression host for lactosylated proteins. To generate Kp and *E. coli* mutant strains, the kanamycin resistance gene *aph* was used to replace the target gene. The resistance cassette replacement constructs were amplified from the pKD4 vector using forward and reverse primers composed of 50 bp homologous to the flanking regions of the gene to be deleted and approximately 20 bp at the 3′ end matching the flanking region of the resistance gene. Primer sequences are listed in Table S2. PCR products were purified and were used to transform recombination-prone cells carrying pKD46 following methods described previously [1]. After gene deletion, the kanamycin resistance gene was removed through FLP-mediated recombination using the pCP20 plasmid to yield markerless mutant strains. Plasmids and protein coding sequences were ordered from GeneArt or Twist Biosciences and codons were optimized for protein expression in *E. coli*. Two expression system for cytoplasmic protein glycoengineering were adopted: one based on the use of two different expression vectors for the expression of carrier protein (pBAD myc/his B vector, Thermo Fisher Scientific) and enzymes (pACYCDuet vector, Novagen), as described previously [2], and a second one based on a single plasmid for the expression of both carrier protein and NGT (pET29b(+) vector, Thermo Fisher Scientific). All plasmids are listed in Table S3. For GMMA production, overnight cultures were diluted in HTMC medium (15 g/L Glycerol, 30 g/L Yeast extract, 0.5 g/L MgSO4, 5 g/L KH2PO4, 20 g/L K2HPO4) to an optical density at 600 nm (OD600) of 0.3 and grown at 30 °C in a rotary shaker for 8 hours using baffled flasks with a liquid to air volume ratio of 1:5. For recombinant protein production, overnight LB cultures were diluted in HTMC medium with 5 g/L glucose instead of 15 g/L glycerol, supplemented with appropriate antibiotics (kanamycin 50 µg/mL, chloramphenicol 20 µg/mL, ampicillin 100 µg/mL). Bacteria were grown for 7.5 h at 30 °C and then protein expression was induced with 0.4% Arabinose and/or with 1 mM IPTG, incubating cultures in baffled flasks (with a liquid to air volume ratio of 1:5) at 25 °C in a rotary shaker for 18 h.

Table S2. List of primers used in this study. Bold regions in the primer sequences correspond to the base pairs matching the flanking regions of the kanamycin resistance gene *aph* in the pKD4 vector.

**Mutated gene**	**Primers**	**Sequence (5' → 3')**	**Mutant strain generated**
tolR	Forward	ACAGGTTCAGCGCTTTAATAATTTCATCGTAAGGGACGTCTTTCGCACCG**GTGTAGGCTGGAGCTGCTTC**	Kp NCTC11228 Δ*tolR*
	Reverse	CATCGTTCCGCTGCTGGATGTCCTGCTGGTGCTGCTGCTGATCTTTATGG**CATATGAATATCCTCCTTAG**	
lacZ	Forward	TCGCCAGCTGGCGTAATAGCGAAGAGGCCCGCACCGATCGCCCTTCCCAA**GTGTAGGCTGGAGCTGCTTC**	*E. coli* K12 W3110 Δ*lacZ*
	Reverse	ATATGGAAACCGTCGATATTCAGCCATGTGCCTTCTTCCGCGTGCAGCAG**CATATGAATATCCTCCTTAG**	

Table S3. List of plasmids used in this study.

**Plasmid**	**Resistance**	**Inducer**
pBAD-*mrkA*	Ampicillin	Arabinose
pBAD-*mrkA* (GlycoTag1)	Ampicillin	Arabinose
pBAD-*mrkA* (GlycoTag2)	Ampicillin	Arabinose
pBAD-*mrkA* (GlycoTag3)	Ampicillin	Arabinose
pBAD-*mrkA* (4xNAT sequon)	Ampicillin	Arabinose
pACYCDuet-*ngt*	Chloramphenicol	IPTG
pACYCDuet-*ngt*+*lgtB*	Chloramphenicol	IPTG

Purification and characterization of recombinant proteins in E. coli

Recombinant proteins were extracted from bacterial pellets stored at -20 °C. Cell lysis was performed either using CelLytic reagent (Sigma-Aldrich) or by sonication (10' for g of biomass). The lysate was then centrifuged to recover the soluble fraction (i.e., the supernatant). The supernatant was filtered and loaded onto HisTrap FF 1 mL or 5 mL (Cytiva) depending on the bacterial growth scale. After sample application, column was washed with 20 mM NaH2PO4, 500 mM NaCl and 20 mM imidazole pH 7.4, then the His-tagged protein was eluted with a linear gradient up to 500 mM imidazole. SDS-PAGE analysis was performed on Immobilized Metal Affinity Chromatography (IMAC) fractions to pool fractions containing the protein of interest. Imidazole was removed from the pooled fractions with Amicon ultrafiltration devices (cut-off 10 kDa for MrkA and sfGFP), and proteins were exchanged in Phosphate-buffered saline (PBS) 1X. Sodium dodecyl sulphate–Polyacrylamide Gel Electrophoresis (SDS–PAGE, 4-12% Bis-Tris gel run in MES buffer) and High-performance Liquid Chromatography–Size Exclusion Chromatography (HPLC–SEC) analyses were performed to check purity of the sample. Samples were analyzed with a TSK gel G3000 PWXL column (30 cm x 7.8 mm; particle size 7 µm; cod. 808021) with TSK gel PWXL guard column (4.0 cm x 6.0 mm; particle size 12 µm; cod.808033) (Tosoh Bioscience).

High Performance Anion-exchange Chromatography with Pulsed Amperometric Detection analysis of glycoproteins

Glycoproteins were hydrolyzed with 2 M trifluoracetic acid (TFA) at 100 °C for 4 h following a method used for the hydrolysis of polysaccharide-containing samples and subsequent quantification of released neutral sugars through High Performance Anion-exchange Chromatography with Pulsed Amperometric Detection (HPAEC–PAD) [3]. After hydrolysis, samples were dried using Speedvac Vacuum concentrator (Thermo Fisher Scientific, Waltham, MA, USA), reconstituted in water, and filtered using 0.45-µm 96-well plate filters for chromatographic analysis. HPAEC–PAD was performed using Dionex ICS5000 system (Thermo Fisher Scientific, Waltham, MA, USA) equipped with a CarboPac PA10 column, 4 × 250 mm (Thermo Fisher Scientific Waltham, MA, USA), coupled to a PA10 guard column, 4 × 50 mm (Thermo Fisher Scientific Waltham, MA, USA). Separation of sugar monomers in the samples was performed eluting in 18 mM NaOH over 20 min at a flow rate of 1 mL/min. The column was then washed for 10 min with 100 mM Sodium Acetate (AcONa), 28 mM NaOH and re-equilibrated with 18 mM NaOH for 20 min. The effluent was monitored using an electrochemical detector in the pulse amperometric mode with a gold working electrode and an Ag/AgCl reference electrode. The resulting chromatographic data were

processed using Chromeleon software 7.2. (Thermo Fisher Scientific, Waltham, MA, USA). Calibration curves were built with a mixture of commercial sugar monomers (Gal, Glc), each in the range 0.5–10 μg/mL. The standard was treated in the same way as the samples. The measured sugar content was converted into molar concentration, which was then used to calculate the Glc or Lac (equivalent to Gal)/protein molar ratio.

Intact Mass analysis

Intact proteins mass analysis was performed by direct flow injection into a Q-Exactive HFX mass spectrometer (Thermo Fisher Scientific) operating at resolution 60,000 at 400 *m/z* in ESI positive ion mode. The sample were desalted by Amicon® Ultra MWCO 10 k and diluted to at a concentration of list 5μM with 0.1% formic acid in H_2O: ACN (70:30 v/v) The full mass spectra were acquired from *m/z* 600 to *m/z* 2,000. The following experimental parameters were applied: flow rate 8μ/min, nitrogen sheat gas 15 a.u., spray voltage 3.5 kV, funnel RF level 200 and capillary temperature 275°C. The obtained mass spectra were then deconvoluted by Biopharma Finder™ 2.0 (Thermo Fisher Scientific).

Protein digestion and LC-MS/MS analysis for peptide mapping

For peptide mapping, about 25 μg of proteins were reduced with 10 mM Dithiothreitol (DTT) and alkylated with 55 mM iodoacetamide (IAA). Trypsin (20 μg/ml) was added. The ratio protein:enzyme used was 20:1. The mixture was incubated overnight at 37° C. The resulting tryptic peptides were desalted with cartridges (Oasis HLB, Waters) and then they were dried under nitrogen flow before the mass spectrometry analysis. The dried samples were resuspended in water and 0.1%TFA. The LC-MS/MS analyses were performed using Q-Exactive HF-X Orbitrap mass spectrometer (Thermo Fisher Scientific) coupled to a liquid chromatographer RLSC Ultimate 3000 (Thermo Fisher Scientific). The chromatographic separation was carried out using a PepMap RSLC C18 column, 75 um × 15 cm, 2 μm, 100 Å (Thermo Fisher) at a flow rate of 300 nl/min. The mobile phases A and B were 0.1% formic acid in water and 0.1% formic acid in acetonitrile, respectively. The starting gradient conditions were 5% of B and 95% A. Phase B increased to 90% in 60 min. The mass spectrometer was set for acquiring in data dependent mode (DDA) able to select the "top ten" most-abundant ions for MS/MS analysis, in ESI positive ion mode.

OAg purification and characterization

O1v1 OAg was extracted from GMMA as previously described [4]. Briefly, GMMA were purified from the 0.22 μm filtered (Stericup filters, Millipore) supernatant culture, after bacteria

centrifugation at 5,000 x g for 45 minutes, through 2 rounds of ultracentrifugation (175,000 x g for 2 hours at 4° C) and finally resuspended in PBS. Acetic acid hydrolysis was used to break the labile linkage between lipid A and KDO at the reducing end of the core region with release of OAg repeats attached to the core in the supernatant. Gel filtration chromatography was used to separate the OAg from core oligosaccharides with no OAg repeats and other impurities. Total extracted OAg was run on a HiPrep 16/60 Sephacryl S100 HR column (600 mm x 16 mm) (GE Healthcare). The mobile phase was PBS at flow rate of 0.5 mL/min. OAg was characterized by HPLC–SEC with differential refractive index (dRI) detection to estimate the molecular size distribution. OAg samples were run on a TSK gel G3000 PWXL column (30 cm x 7.8 mm; particle size 7 µm; cod. 808021) with TSK gel PWXL guard column (4.0 cm x 6.0 mm; particle size 12 µm; cod.808033) (Tosoh Bioscience). The mobile phase was 0.1 M NaCl, 0.1 M NaH_2PO_4, 5% CH_3CN, pH 7.2 at the flow rate of 0.5 mL/min (isocratic method for 35 min). OAg peak molecular mass (MP) was calculated using dextrans as standards in the range 12 - 150 kDa. Sugar content was estimated by High-performance Anion-exchange Chromatography with Pulsed Amperometric Detection (HPAEC–PAD) analysis[3]. Nuclear Magnetic Resonance (NMR) spectroscopy was used to confirm PS identity and purity. All NMR experiments were performed with a Bruker Advance 800 MHz spectrometer equipped with a high-precision temperature controller using a 5 mm QCI CryoProbe. Spectra were weighted with 0.8 Hz line broadening and Fourier-transformed. NMR spectra were recorded at 50.0 ± 0.1 °C. The transmitter was set at the water frequency (4.70 ppm). Suppression of the water signal was achieved by excitation sculpting (2 ms selective square pulse). Proton spectra were acquired using a 90-degree pulse duration automatically calculated and collected with 32K data points over a 12 ppm spectral width, accumulating 128 number of scans. Spectra were processed by applying an exponential function to the FID with a line broadening of 0.80 Hz to increase the signal-to-noise ratio and then Fourier transformed. Data acquisition and processing were performed with TopSpin 3.5 software package (Bruker BioSpin).

KAg purification and characterization

K2 was extracted directly from Kp strain NCTC11228 by acetic acid hydrolysis. It was then purified through fractional precipitation with CTAB as previously reported [5]. K2 was characterized by HPLC–SEC with dRI detection to estimate the molecular size distribution, using a TSK gel G3000 PWXL column (30 cm x 7.8 mm; particle size 7 µm; cod. 808021) with TSK gel PWXL guard column (4.0 cm x 6.0 mm; particle size 12 µm; cod.808033) (Tosoh Bioscience). K2 peak molecular mass (MP) was calculated using dextrans as standards in the range 50 - 410 kDa. Sugar content was estimated by High-performance Anion-exchange Chromatography with Pulsed

Amperometric Detection (HPAEC–PAD) analysis [3]. DNA and protein impurities were determined by measuring absorbance at 260 nm and through microBCA, respectively. Placing K2 in an ice bath, 30 cycles of sonication (VibraCell, Sonics and Materials Inc.) were performed to reduce its size (resulting PS is indicated as sK2). The sonication cycle consisted of 30’’ of pulses followed by 30’’ of rest. NMR spectroscopy was used to confirm PS identity and purity, as for OAg characterization.

Synthesis and purification of selective MrkA glycoconjugates

OAg and KAg were conjugated to Lac-MrkA using the following selective approach. PS were activated with CDAP and subsequently derivatized with adipic acid dihydrazide (ADH). Briefly, OAg (at 5 mg/mL in water) or K2 (2 mg/mL in water) was placed in an ice bath and the solution brought to pH 9. Reaction with CDAP (w/w ratio CDAP/PS of 0.2 for OAg and of 1 for K2) was performed at 0 °C for 15 min under stirring, as previously reported [6]. After 15 min, 0.25 M ADH was added, and reaction mixture was kept in agitation for 2 h at room temperature. Derivatized sugars were purified with PD10 Desalting column prepacked with Sephadex G-25 Superfine (GE Healthcare) to remove excess of free ADH. Conjugation reaction was performed via reductive amination between randomly ADH-derivatized PS and oxidized lactose moiety on MrkA. After protein concentration (15-20 mg/mL) and buffer exchange in 100 mM Acetate pH 4.5, Lac-MrkA was oxidized with 5 mM sodium periodate (NaIO4) for 30’ at 25 °C. After that, reaction was quenched with 10 mM Na2SO3 for 15’ at room temperature on a rotating wheel. Oxidized protein was added to lyophilized PS-ADH (2:1 w/w protein to PS for K2, 1:1 w/w for OAg), $NaBH_3CN$ (5 molar excess respect to reactive aldehydes) was added for imine reduction and left ON at room temperature on a rotating wheel. Conjugation mixtures were purified through a first step of Size Exclusion Chromatography with HiPrep 16/60 Sephacryl S100 HR column (Cytiva), for O1v1 conjugate, or HiPrep 16/60 Sephacryl S300 HR column (Cytiva), for K2 conjugate, to remove unconjugated protein. Isocratic Elution at 0.5 mL/min in PBS 1x was used. Pooled SEC fractions containing the conjugate were then loaded onto a HisTrap Fast Flow 1 mL column (Cytiva) for the affinity purification of the conjugate taking advantage of the HisTag present on the protein, for the removal of the eventual free saccharide. The column was equilibrated and washed after sample application with 20 mM NaH_2PO_4, 500 mM NaCl pH 7.4, conjugate was then eluted with 500 mM of imidazole. Imidazole was removed using Amicon centrifugal filters (cut-off 30 kDa) and exchanged again in PBS 1X. Flow-through and elution pools were characterized by microBCA to verify presence of the conjugate in the eluates.

Glycoconjugates characterization

Intermediates of conjugation were characterized as previously described [7]. Purified conjugates were characterized by HPAEC–PAD analysis for sugar quantification and by microBCA for protein quantification, using BSA as a reference following the manufacturer's instructions (Thermo Fisher Scientific); the ratio of saccharide to protein was then calculated. HPLC–SEC was used to verify conjugates formation and detect the presence of unreacted protein [7]. OAg glycoconjugates were run on a TSK gel G3000 PW XL column (Tosoh Bioscience), while KAg glycoconjugates on TSKgel G6000PW XL and G5000PW XL columns (Tosoh Bioscience) connected in series.

SDS-PAGE/western blot analyses were performed on SEC fractions and on final purified OAg conjugates. In brief, samples were first mixed with LDS Sample Buffer supplemented with reducing reagent (Thermo Fisher Scientific) and heated at 100 °C for 3 min. Heated samples were then resolved by a 4%–12% Bis-Tris SDS-PAGE gel (Thermo Fisher Scientific). For SDS-PAGE gel staining, the gel was stained with ProBlue Safe Stain (Giotto Biotech) for 2 h at room temperature with agitation, followed by three washes with deionized water to remove excess dye. The stained gel was then imaged by a CCD camera under white light (Chemidoc Imaging System, Bio-Rad). For Western blot, the resolved proteins were transferred from the SDS-PAGE gel onto a PVDF membrane using the iBlot (Thermo Fisher Scientific) according to the manufacturer's instructions. The membrane was then blocked in 10% non-fat milk (diluted in 1X PBS 0.05% TWEEN 20) for 1 h at room temperature and sequentially incubated with primary antibodies and HRP-conjugated secondary antibodies, both for 1 h at room temperature. Following extensive washes with 1X PBS 0.05% TWEEN 20, the membrane was incubated with luminol-based chemiluminescent substrate (Thermo Fisher Scientific), and the immuno-bands were captured by a CCD camera (Chemidoc Imaging System, Bio-Rad). The following primary antibodies were used: mouse anti-His (1:2000, Qiagen), mouse anti-O1v1 mAb (KPE33, 1:2000), and mouse anti-MrkA mAb (KP3, 1:2000). As secondary antibody, rabbit anti-mouse IgG-HRP was used (1:5000). All antibodies were diluted in 3% non-fat milk 1X PBS 0.05% TWEEN 20.

Immunogenicity studies in animal models

Animal studies were performed at GSK Animal Resources Centre in Siena under the animal project 479/2017-PR 09/06/2017 approved by the Italian Ministry of Health. All animal studies were ethically reviewed and carried out in accordance with European Directive 2010/63/EEC and the GSK policy on the Care, Welfare and Treatment of Animals. In the mice study with MrkA conjugates, 10 female CD1 mice (4-6 weeks old) were SC immunized at day 0 and 28 at 5 µg MrkA

dose formulated in Alhydrogel (Aluminium hydroxide at 0.7 mg/mL $Al3+$), while in the study in rabbits, 8 female New Zealand White rabbits were immunized intramuscularly at day 0 and 28 at 15 µg MrkA dose formulated in Alhydrogel (Aluminium hydroxide at 0.7 mg/mL $Al3+$). Sera were collected at days 27 and 42. Individual animal sera were tested for anti-MrkA, anti-O1v1 and/or anti-K2 total IgG by enzyme-linked immunosorbent assay (ELISA) as previously described [8]. MrkA at a concentration of 15 µg/mL, O1v1 at 0.6 µg/mL and K2 at 15 µg/mL in PBS pH 7.4 were used as coating antigens. Results are reported as anti-antigen specific IgG EU/mL: one ELISA unit is defined as the reciprocal of the standard serum dilution that gives an absorbance value equal to 1. Bars in the graph represent geometric mean titers and each dot results from each single animal. SBA analysis against Kp strain NCTC11228 (K2:O1v1) was performed as previously reported [9]. Results of the assay were expressed as the IC50, the reciprocal serum dilution that resulted in a 50% reduction of luminescence and thus corresponding to 50% growth inhibition of the bacteria present in the assay. GraphPad Prism software (GraphPad Software, La Jolla, CA, USA) was used for curve fitting and IC50 determination. A titer equal to half of the first dilution of sera tested (10) was assigned to not bactericidal sera.

Flow cytometry

Kp strain NCTC11679 (K61:O3) was grown ON in LB medium at 37 °C and 5% $CO2$. Bacteria were pelleted at 4000 × g for 5 min and washed with PBS. Bacteria were then blocked with PBS + 3% (w/V) BSA for 20 min and incubated with pooled mice or rabbit sera diluted 1:500 in PBS + 1% (w/V) BSA for one hour. After washes, samples were incubated with FITC anti-mouse IgG (Thermo Fisher Scientific, Waltham, MA, USA) diluted in PBS + 0.1% (w/V) BSA for 45 min at 4°C. Finally, bacteria were fixed with 4% (w/V) formaldehyde for 20 min. Flow cytometry analyses were performed on a FACS Canto II flow cytometer (BD Biosciences). Results are reported as overlaid histograms with the relative fluorescence intensity on the X axis and the percentage of the maximum number of events on the Y axis.

Statistical analysis

Statistical analysis was performed using GraphPad Prism 7 (GraphPad Software, La Jolla, CA, USA). The non-parametric Mann-Whitney two-tailed test and Kruskal-Wallis analysis with post-hoc Dunn’s test were used respectively to compare two or multiple groups. Wilcoxon matched-pairs signed rank two-tailed test was used to compare results from the same group at different time points.

References

1. K. A. Datsenko and B. L. Wanner, *Proceedings of the National Academy of Sciences*, 2000, **97**, 6640.
2. H. L. P. Tytgat, C. W. Lin, M. D. Levasseur, M. B. Tomek, C. Rutschmann, J. Mock, N. Liebscher, N. Terasaka, Y. Azuma, M. Wetter, M. F. Bachmann, D. Hilvert, M. Aebi and T. G. Keys, *Nat Commun*, 2019, **10**, 5403.
3. F. Micoli, S. Rondini, M. Gavini, L. Lanzilao, D. Medaglini, A. Saul and L. B. Martin, *PLoS One*, 2012, **7**, e47039.
4. G. De Benedetto, R. Alfini, P. Cescutti, M. Caboni, L. Lanzilao, F. Necchi, A. Saul, C. A. MacLennan, S. Rondini and F. Micoli, *Vaccine*, 2017, **35**, 419-426.
5. F. Nonne, M. Molfetta, R. Nappini, C. La Guidara, R. Di Benedetto, S. Mfana, B. Bellich, M. M. Raso, G. Gasperini, R. Alfini, P. Cescutti, F. Berlanda Scorza, N. Ravenscroft, F. Micoli and C. Giannelli, 2024, **13**, 256.
6. R. Nappini, R. Alfini, S. Durante, L. Salvini, M. M. Raso, E. Palmieri, R. Di Benedetto, M. Carducci, O. Rossi, P. Cescutti, F. Micoli and C. Giannelli, 2024, **12**, 707.
7. G. Stefanetti, S. Rondini, L. Lanzilao, A. Saul, C. A. MacLennan and F. Micoli, *Vaccine*, 2014, **32**, 6122-6129.
8. L. Lanzilao, G. Stefanetti, A. Saul, C. A. MacLennan, F. Micoli and S. Rondini, *PLOS ONE*, 2015, **10**, e0139847.
9. F. Nonne, M. Molfetta, G. F. Belciug, M. Carducci, V. Cianchi, C. Zakroff, S. Durante, C. Zellmer, S. Baker, T. D. Stanton, K. E. Holt, K. Wyres, N. Ravenscroft, G. Gasperini, O. Rossi, C. Giannelli, F. Berlanda Scorza and F. Micoli, *Communications Biology*, 2025, **8**, 898.
